# Kinetics *versus* thermodynamics in the proline catalyzed aldol reaction†
†Electronic supplementary information (ESI) available: Experimental details, calculated energies, geometries and simulations. See DOI: 10.1039/c6sc01328g


**DOI:** 10.1039/c6sc01328g

**Published:** 2016-05-06

**Authors:** M. Orlandi, M. Ceotto, M. Benaglia

**Affiliations:** a Dipartimento di Chimica , Università degli Studi di Milano , via C. Golgi, 19 , 20133 Milano , Italy . Email: michele.ceotto@unimi.it ; Email: maurizio.benaglia@unimi.it

## Abstract

In this paper the equilibrium properties of the proline catalyzed aldol reaction was studied. The use of well-established methodologies, like reaction progress kinetic analysis and linear free energy relationship analysis, led to the quantification of the reaction reversibility and to its correlation with the substrate electronic activation. Due to these experimental observations, common computational approaches based on a one way transition state analysis become unsuitable. Therefore, a computational model based on the integration of a system of kinetic differential equations associated to the multiple equilibrium reactions was proposed. Such a model was found to successfully rationalize the chemical and stereochemical outcomes of this paradigmatic reaction for the first time.

## Abstract 2

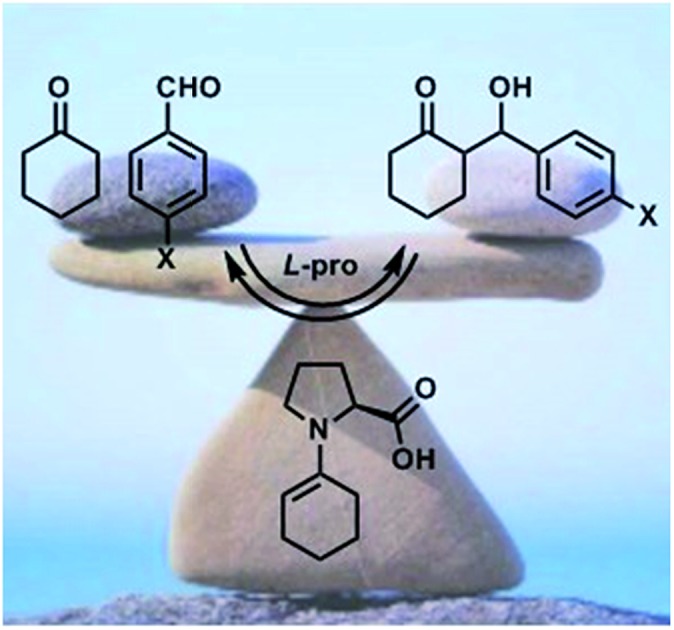
The reversibility of the reaction was proved and a new rate law was obtained; the use of a Multiple Transition State Approach (MTSA), that includes both kinetics and thermodynamics in the description of the process, successfully rationalizes the chemical and stereochemical outcomes of this paradigmatic reaction for the first time.

## Introduction

After the pioneering works by List and Barbas,^1^ Jacobsen,^2^ and MacMillan,^3^ organocatalyzed reactions have been studied through experimental techniques and theoretical methods to rationalize the stereochemical behavior of a great number of catalysts.^4^ Among the others, Blackmond, List and Houk have extensively investigated the intra- and inter-molecular proline catalyzed aldol reactions of ketones and aldehydes.^5^,^6^ In particular, by using Reaction Progress Kinetic Analysis (RPKA), Blackmond *et al.* studied the proline catalyzed addition of acetone to 2- and 3-chlorobenzaldehyde, defining for the first time the kinetic rate law of this important reaction.5a–c Earlier, Houk and List provided the first evidence of the reaction mechanism, which involves the stereoselectivity model commonly known as the Houk–List model.^6^ Despite first reports about proline catalysis going back to more than fifteen years ago, new efforts are still ongoing from researchers in order to improve the knowledge about the nature of this kind of reaction and to better explain in detail some aspects of these transformations.^7^

Here we report a study about the equilibrating nature of this reaction and about the influence of such reversibility on its stereochemical outcome. Eventually, this study leads to the application of a multiple transition state approach for the first quantitative computational rationalization of the observed yields, dr and ee.

## Results and discussion

**Scheme 1:**
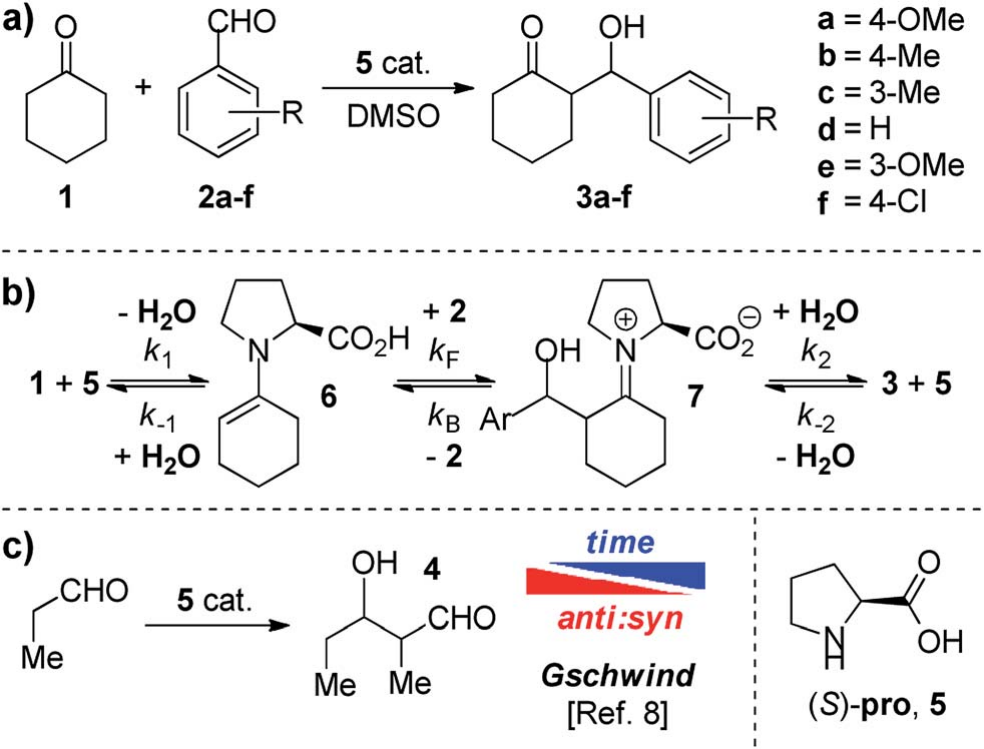
(a) Reactions studied in the present work. (b) Enamine formation pre-equilibrium and reversible addition of enamine **6** to the aldehyde **2**. (c) Observed dependence of diastereoselectivity with time by Gschwind *et al.*

In this work, we focused our attention on the proline catalyzed addition of cyclohexanone **1** to several benzaldehydes **2a–2f** (Scheme 1a). In particular, we wondered about the reversibility of these reactions accordingly with Scheme 1b. Indeed, NMR studies by Gschwind *et al.*^8^ have clearly shown the time dependence of the *syn* : *anti* ratio of aldol **4** in the presence of (*S*)-proline **5** (Scheme 1c), which is a symptom of reversible processes.

**Scheme 2:**
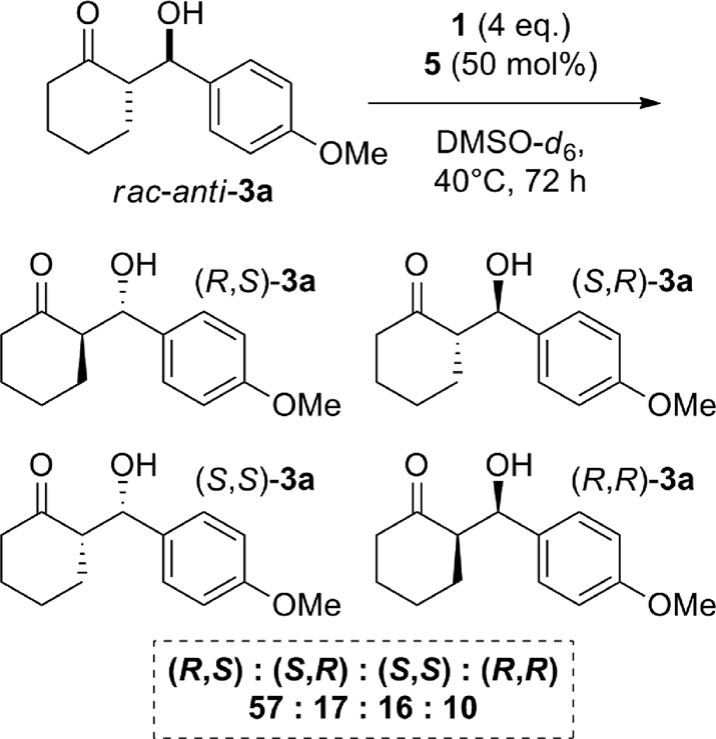
Kinetic resolution of racemic *anti*-**3a** in the presence of **5**.

By monitoring racemic *anti* ketols **3a**, **3d** and **3f** in the presence of proline in DMSO-*d*_6_ through ^1^H NMR techniques, we observed the delivery of the corresponding aldehydes (see Table S1, ESI† for additional details). In order to determine the dependence of the retro aldol reaction on the proline, we mixed racemic *anti*-**3a** with **5** (30 mol%) and cyclohexanone **1** (4 eq.) in DMSO-*d*_6_ and the appearance of *syn*-**3a** was detected (Scheme 2). In particular, the CSP-HPLC analysis of the crude mixture after 72 h revealed a 1 : 3 *syn* : *anti* ratio and 53% ee for the (*R*,*S*) enantiomer of *anti*-**3a**, proving a kinetic resolution of the racemic starting material (Scheme 2) as a clear indication of the reversibility of the reaction.

The effect of this equilibration in the proline catalyzed aldol reaction has already been detected,^8^ yet, it has never been studied in detail. Hence, in the present work, we will employ well-established methodologies, such as RPKA, LFERs (Linear Free Energy Relationships) and DFT computational methods to investigate this equilibrating phenomenon and rationalize its effect on the reaction outcome.

The RPKA approach, developed by Blackmond, represents one of the most powerful tools for the study of reaction mechanisms.^9^ It has been also used for the clarification of the role of water in the proline catalyzed aldol reaction5*b* and for the determination of the power rate law for the addition of acetone to 3-chlorobenzaldehyde.5*a* Such a reaction was found to proceed up to complete conversion and it is very well described by the following equation:5*a*

1 Rate = *k*[ketone]^0.59^[aldehyde]^0.9^[H_2_O]^–0.7^

Eqn (1) represents the current state of the art equation and it is an excellent description for aldol reactions that provide near complete conversions. Given the reaction of acetone with 3-chlorobenzaldehyde, a rate law that considers reversibility was not required in those cases, and the possibility of reverse reactions was not included in eqn (1).5*a* However, in our specific cases, eqn (1) cannot be applied straightforwardly due to the presence of a substantial retro aldol reaction.

**Fig. 1:**
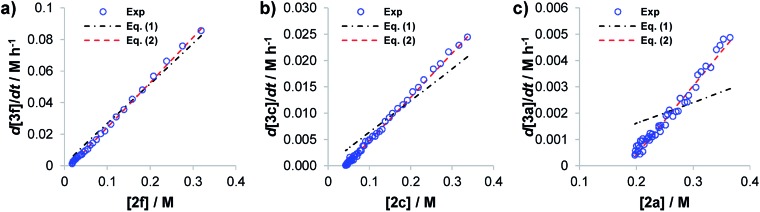
“d[**3f**]/d*t vs.* [**2**]” graphical rate laws for aldehydes (a) **2f**, (b) **2c** and (c) **2a**. Blue circles: experimental. Dashed black lines: fitting with eqn (1). Dashed red lines: fitting with eqn (2).

This can be observed through the “graphical rate laws” reported in Fig. 1. Fig. 1a shows the rate law for the electron poor aldehyde **2f**. Fig. 1b is the same plot but for aldehyde **2c**, which presents an intermediate reactivity, and Fig. 1c is for the poorly reactive aldehyde **2a**. From a direct comparison of the three graphs, it is clear that the ability of eqn (1) to fit the reaction progress fails as the reaction conversion decreases (see the black dashed lines in Fig. 1a–c for the data fitting with eqn (1)). This observation motivated us to improve eqn (1) into a new rate law that includes reversible processes.

To reach such a goal, we have chosen ^1^H NMR as the analytical technique because it allows monitoring of the concentration of reagents and products, as well as diastereoselectivity, *versus* time. The reaction between cyclohexanone **1** and 4-chlorobenzaldehyde **2f** was chosen as a benchmark reaction, since it provides higher reaction rates with respect to aldehydes **2a–2e**. Firstly, a “same excess”^9^ experiment was performed by running two reactions at different concentrations but with the same catalyst amount and the same excess of **1** with respect to **2f**. This experiment highlighted the absence of catalyst deactivation in the presence of water 0.73 M (see Fig. S4, ESI†), in accordance with previous observations by Pihko5p,q and rationalization by Blackmond.5b,c Then “different excess”^9^ experiments were run with [exc] = 0.0, 0.24, 0.93, 1.53 and 2.6 M. Interestingly, we found that a good description of the reaction kinetics can be obtained only by considering the overall process that involves not only the forwards addition of enamine **6** to the aldehyde **2**, but also the backwards aldol reaction that takes place by interaction of the catalyst **5** with the product **3** (Scheme 1b). Moreover, pre-equilibrium kinetics for the formation of enamine **6** was accounted for to rationalize the observed dependence of the reaction rate on [**1**] and [H_2_O] (Fig. S5, ESI†).

The observed first order dependence of the retro aldol reaction on **3** was confirmed through additional RPKA experiments. Indeed, by monitoring the retro aldol reaction of **3a** and by plotting the disappearance rate of **3a** (–d[**3a**]/d*t*) against [**3a**], a linear dependence was observed. This behavior has been rationalized by assuming a large dissociation constant (*k*_2_/*k*_–2_) for **7** (see page S9, ESI†).

Eventually, we derived the following rate law and a detailed derivation of these equations can be found on pages S5–S9 of the ESI.†
2
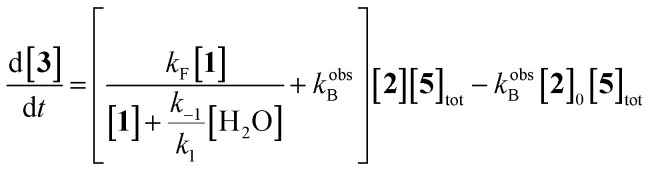

3
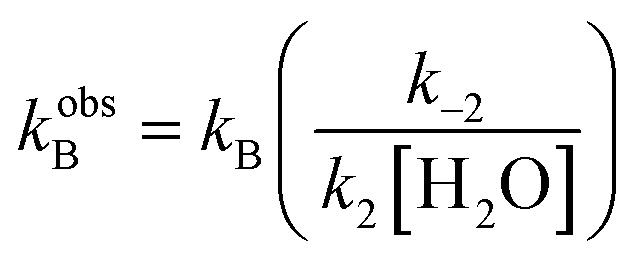



It is important to note that eqn (2) represents an extension of the Blackmond's power law (1), where the reversible process has been included. By putting *k*obsB equal to zero, eqn (1) is recovered. Eqn (2) allows us to reproduce the experimental reaction progress curves even for those aldehydes with lower reactivity (see Fig. 1a–c, red dashed lines).

After proving that eqn (2) provides a complete kinetic description of the catalytic system, we acquired reaction profiles of the reactions involving aldehydes **2a–2f**.

The presence of different substituents at the 3- or 4-position on the phenyl ring of the aldehyde are not expected to effect this aldol reaction *via* steric interactions. However, the modulation of the electron-withdrawing/electron-donating properties of such substituents can strongly affect the activation and the reactivity of the carbonyl moiety. Indeed, from the experimental plots reported in Fig. 1, a strong dependence of the reaction's profile, and more surprisingly, of the final reaction conversion on the aldehyde substituent is evident. In Fig. 1 the linear dependence of the rate to equilibrium on the aldehyde concentration (d[**3**]/d*t vs.* [**2**]) is highlighted. Note that the non-zero intercepts, especially in Fig. 1c, are a clear indication of equilibration, since the final conversions of the reactions are not quantitative (Fig. S8 of the ESI† reports the experimental profiles for all of the aldehydes).

The kinetics study hereby presented is evidence of the dependence of the reaction on the electronic properties of the substrate **2**. Thus, for a better rationalization of these observations and to provide a quantitative analysis of the observed electronic effects, we looked at the Hammett equation.^10^ Indeed, while the dependence of both reaction rate and reaction conversion on the aldehyde's electronic properties has been qualitatively highlighted in Fig. 1, a quantitative description can be provided by correlating the amounts ln(*k*_X_/*k*_H_) and ln(*K*_X_/*K*_H_)^11^ against *σ*^+^ (the Hammett electrophilic constant10*d*).

**Fig. 2:**
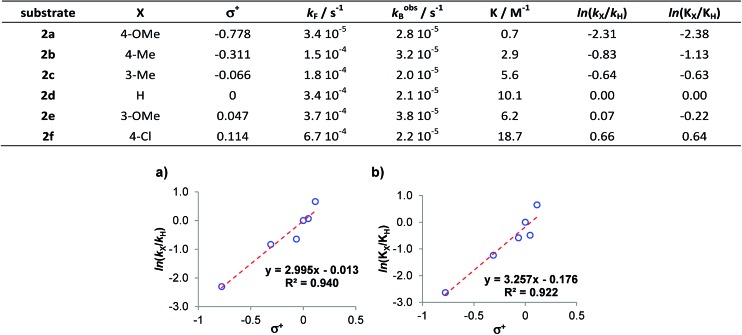
(a) Correlation between the relative reaction rates *k*_X_/*k*_H_ and the Hammett electrophilic constants *σ*^+^. (b) Correlation between the relative equilibrium constants *K*_X_/*K*_H_ and the Hammett electrophilic constants *σ*^+^.

The plots obtained are reported in Fig. 2, and demonstrate a good correlation. Here an intercept near to zero and an angular coefficient *ρ* of *ca.* 3 were found in both cases. This same dependence of ΔΔ*G*_act_ (or ln(*k*_X_/*k*_H_)) and ΔΔ_R_*G* (or ln(*K*_X_/*K*_H_)) on *σ*^+^ suggests that the only variation in the energetic profile of the reaction is the relative energy level of the ground state reagents. Further support for this observation can be given by a direct comparison of the measured rate constants *k*_F_ and *k*obsB (Fig. 2): while *k*_F_ strongly depends on the aldehyde's activation, *k*_B_ presents a roughly constant value. This means that, intuitively, while different aryl rings affect the reactivity of the aldehyde's carbonyl group, they do not strongly affect the reactivity of the ketols' **3a–3f** carbonyl group. In conclusion, increasing the reactivity of the aldehyde (*i.e.* by increasing its HOMO energy) affects both the rate constant *k*_F_ and the equilibrium constant *K*.

**Fig. 3:**
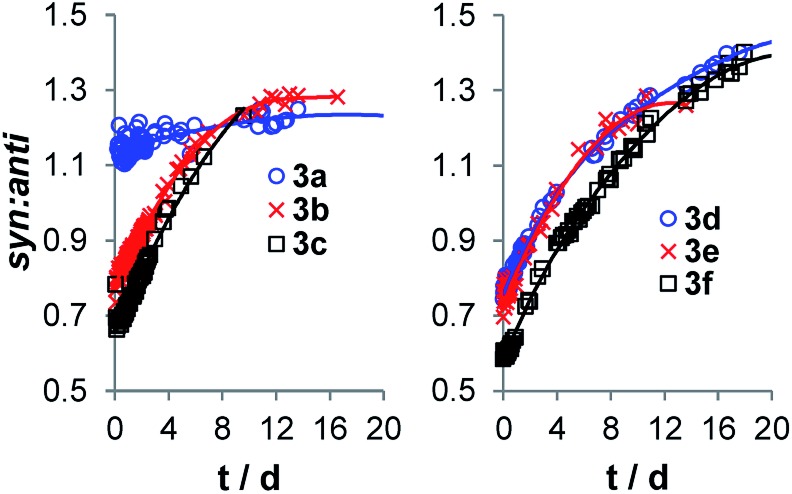
Evidence of epimerization during reaction time.

Most information about the processes affecting a stereoselective chemical reaction can be found by monitoring the isomer distribution. The use of ^1^H NMR as a detection technique for the obtainment of the reaction profiles allows us to monitor the *syn* : *anti* ratio as a function of the reaction time. Gschwind *et al.* already reported the epimerization of diastereomeric products in the proline catalyzed auto-addition of propionaldehyde (Scheme 1c).^8^ For the present case, the *syn* : *anti* ratios of compounds **3a–3f** are plotted against time in Fig. 3. In the graphs the epimerization from the *anti* to the *syn* diastereoisomer for ketols **3b–3f** is evident, where the *syn* : *anti* ratios change from *ca.* 40 : 60 to 58 : 42. Thus, the *anti* isomer is the kinetic product, as correctly predicted by the Houk–List model,^6^ whereas the *syn* isomer is the thermodynamic one. The presence of epimerization is consistent with the retro aldol reaction, and once the reaction equilibrates, the dr will change from the kinetic towards the most stable thermodynamic product.

Interestingly, we found that ketol **3a** exhibits a constant dr. We attribute this phenomenon to the very similar values of *k*_F_ and *k*_B_ for the aldol reaction of **2a** (Fig. 2). Indeed, since the forward and backward processes proceed with similar rates, the epimerization and the aldol addition occur simultaneously and at a comparable rate.

As expected, enantioselectivity is also affected by equilibration. The ee of the six monitored reactions have been evaluated after 6 d of reaction and then after 3 weeks. In Table 1 the obtained results are reported. From these data the following observations are summarized: (i) all the products are affected by racemization, (ii) products characterized by electron-donating groups usually lead to higher ee erosion, and (iii) *anti* diastereoisomers undergo faster racemization than *syn* ones.

**Table 1:** Measured enantioselectivities and evidence of racemization during reaction time

Substrate	*t* (d)	Conv.^*a*^ (%)	dr^*b*^	ee^*c*^ (%)	
				*syn*	*anti*
**2a**	6	58	55 : 45	59	76
	21	68	55 : 45	34	48
**2b**	6	87	53 : 47	73	79
	21	87	56 : 44	54	54
**2c**	6	90	51 : 49	64	60
	21	90	56 : 44	41	19
**2d**	6	95	53 : 47	77	77
	21	95	58 : 42	69	60
**2e**	6	95	53 : 47	82	83
	21	95	58 : 42	72	63
**2f**	6	98	48 : 52	87	84
	21	98	58 : 42	84	78

^*a*^Evaluated through ^1^H NMR analysis of the crude product.

^*b*^Expressed as *syn* : *anti* ratio.

^*c*^Evaluated through CSP-HPLC analysis.

Thus, the stereochemical analysis of the system supports the evidence of a substantial and specific equilibration in accordance with our preliminary experiments (Scheme 2) and kinetic analysis.

Since the observed equilibration has a strong effect on the stereochemical outcome of the reaction, we wondered whether this feature might be responsible for the missing computational rationalization of these reactions. Indeed, although many papers have been published^4^,5e,i,j,n,o,^6^ that qualitatively rationalize the stereochemical outcome of intra and intermolecular proline catalyzed aldol reactions, the quantitative prediction of such a reaction outcome is still an open issue. Rzepa *et al.*7*a* have recently revisited the proline catalyzed addition of **1** to aldehyde **2d** in a comprehensive paper, where the inadequacy of DFT methods in the evaluation of the weak interactions occurring at the TS level is ascribed to be the main responsible factor of such limitations. One can resort to the Curtin–Hammett Principle (CHP),^12^ which has been extensively employed in the determination of the stereoselectivity of organic reactions to better understand these reactions. However, it is well known that this approximation is applicable only if (i) rapidly interconverting reagents, such as conformers, are involved and (ii) the considered processes lead irreversibly to the products.^13^ Hence, its application should be limited only to irreversible processes affording non-interconverting products. The experiments here reported have highlighted the equilibrating nature of the proline catalyzed aldol reaction. Given such dynamic nature, which depends on the electronic nature of the aldehyde, as shown in this study, traditional transition state approaches can no longer be applied.

**Scheme 3:**
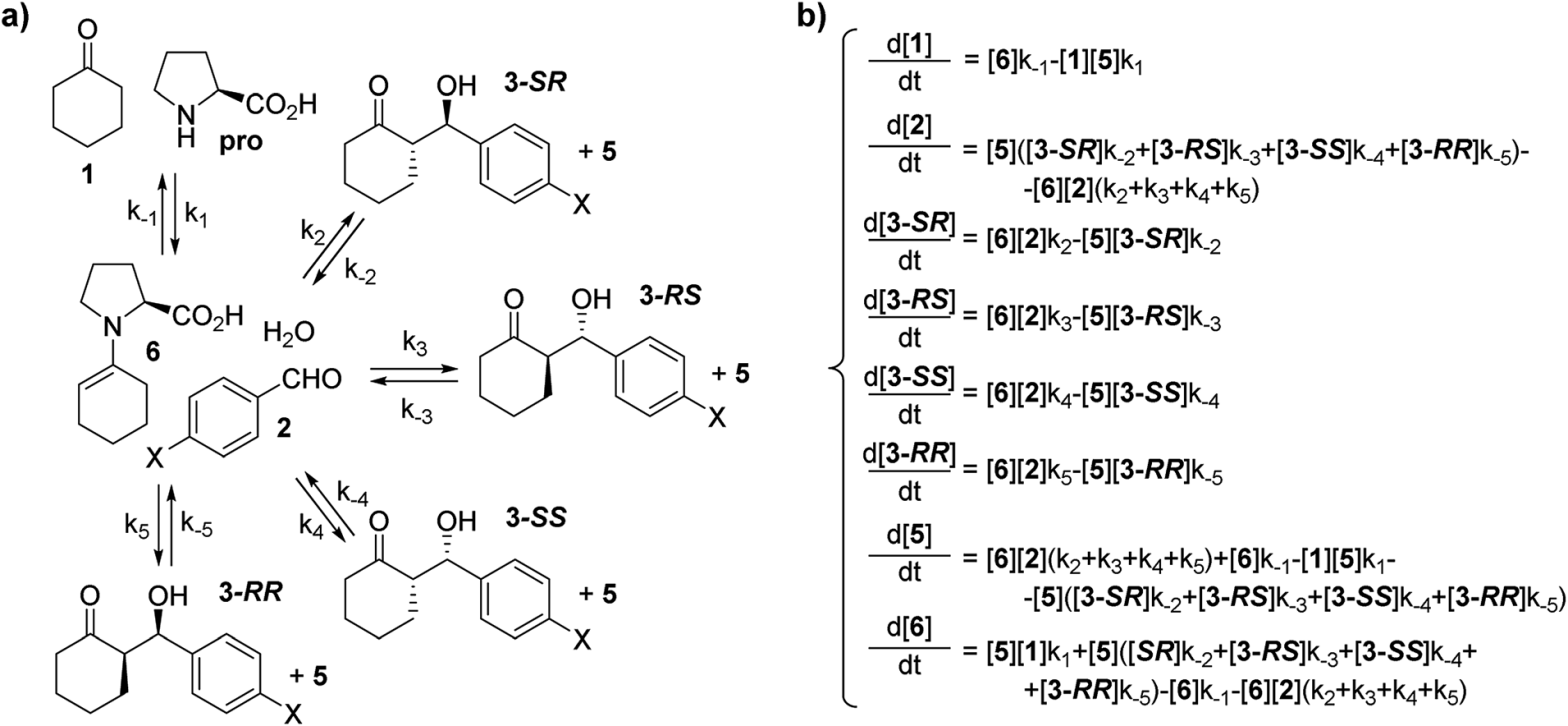
(a) Simplified reaction scheme. (b) System of differential equations associated to the reaction scheme.

Thus, in order to include both kinetics and thermodynamics in the description of the catalytic system, an alternative theoretical approach for the description of the reactivity is required. In particular, we propose that the reaction evolution can be obtained by integrating the rate law of the simplified reaction scheme reported in Scheme 3. More specifically, by calculating the rate constants of Scheme 3a and by numerically integrating the system of differential equations reported in Scheme 3b, a time-dependent picture of product concentration is obtained. Scheme 3a is a simplification of the reaction based on the considerations that: (i) the inclusion of additional species that are in fast equilibrium with the considered compounds (such as water, oxazolidinones and iminium ions) is considered a higher order precision level of calculation, and that (ii) the considered species are the prevalent compounds that characterize the kinetics of the reaction in the presence of water, which is known to suppress side reactions ascribed to the catalyst's deactivation.5b,c In addition, we indeed consider that water has a fundamental role, but its concentration is constant during the reaction. Hence, when considering the qualitative and relative behavior of different reactions involving different aldehydes under the same reaction conditions, it can be neglected. Finally, in Scheme 3b we consider enamine **6** both as a single conformer (s-*trans*) and as a mixture of two conformers (s-*trans* and s-*cis*), each one leading to a couple of product diastereoisomers and we found that this does not affect the final outcome of the simulation.^14^

The numerical resolution of complicated systems of differential equations is commonly known in the field of chemical kinetics.^15^ However, to the best of our knowledge, this is the first time that it has been applied to the field of organic computational chemistry as a tool for the simulation of stereoselective reaction outcomes, going beyond a static single transition state picture. It is important to recall that one can recover the CHP result as the time zero approximation of the present approach, while the infinite time approximation represents the thermal distribution of reagents and products. Since, as just highlighted, both kinetics and thermodynamics play an important role in this reaction, it is important to find a DFT functional capable of evaluating at best the Δ_R_*G*, the Δ*G*^‡^ and the ΔΔ*G*^‡^ values. We focused on the M06/2X functional, since Houk *et al.*^16^ and Hubin and coworkers^17^ showed that it gives the best thermodynamic description for this class of reactions. After a wide screening of several computational set ups, we identified M06-2X/6-311G(2d,2p) as the best compromise for fast and accurate computations of a huge number of structures. More than thirty computational set ups have been tested (Table S6, ESI†) including B3LYP, CAM-B3LYP, mPW1PW91, wB97XD, M06, M06L, M06HF, M052X, BMK and LC-wPBE. Several polarizable continuum models for the inclusion of the solvent effect have also been tested, but these usually provided endorgonic profiles (Δ_R_*G* > 0). Thus, the structures of the species depicted in Scheme 3a and the TSs connecting the involved processes have been computed for aldehydes **2a–2f**, in compliance with the extended conformational analysis reported by Rzepa *et al.*7*a* In Table 2 the calculated Δ_R_*G* values for both *syn*- and *anti*-**3a–3f** and the Δ*G*^‡^ values for the pathway involving the favourite TS (leading to **3-SR**) are reported for all the substrates.

**Table 2:** Computed Δ*G*^‡^ for TSs involving **2a–2f**, and Δ_R_*G* for both *syn*- and *anti*-**3a–3f**^*a*^

Substrate	Δ*G*^‡^	Δ_R_*G*	
		*syn*	*anti*
**2a**	20.1	0.24	0.57
**2b**	18.8	–1.95	–0.51
**2c**	18.6	–0.84	–0.68
**2d**	18.7	–1.42	–0.62
**2e**	18.9	–1.62	–0.44
**2f**	18.7	–2.45	–1.47

^*a*^All the energy values reported are in kcal mol^–1^.

From the values reported in Table 2, it can be observed that computations are only partially able to predict the experimentally observed trend. Indeed, while **2a** is correctly predicted to be the least reactive aldehyde (Table 2), the other substrates were calculated to have roughly the same reactivity (Table 2). However, a quite good description of Δ_R_*G* was found, since on increasing the electron-withdrawing character of the aldehyde substituent, the product stability becomes higher (with a few exceptions).

The rate constants required to obtain the system of differential equations in Scheme 3b, were calculated by means of transition state theory.^18^ According to the Eyring equation, rate constants with an order of magnitude of 10^2^ s^–1^ are obtained with calculated energy barriers of *ca.* 19 kcal mol^–1^. However, the experimentally obtained values for *k*_F_ have a magnitude of 10^–4^ s^–1^. Thus, we may expect a substantial error in the time scale when simulating these reactions which might be considerably shorter. Nevertheless, the simulations should in principle still maintain a good relative description of the reactions including the reversibility of the system, especially when compared with the simple TS analysis.

The numerical integration of the equations in Scheme 3b, under adequate boundary conditions (in compliance with the experimentally used reagent concentrations), was performed by means of the ODE15s algorithm provided by MatLab suite of codes.^19^ In Table 3 the results obtained with this Multiple Transition State Approach (MTSA) and with the CHP are reported for the reactions relative to aldehydes **2a–2f**.

**Table 3:** Simulated reaction outcome within the CHP and with the MTSA^*a*^

Substrate	Multiple transition state approach					Curtin–Hammett principle		
	*t* (h)	Conv. (%)	dr^*b*^	ee (%)		dr^*b*^	ee (%)	
				*syn*	*anti*		*syn*	*anti*
**2a**	34	68	66 : 34	62	79	15 : 85	99	>99
	95	73	66 : 34	30	53			
**2b**	40	93	58 : 42	71	85	6 : 94	99	>99
	105	94	60 : 40	43	66			
**2c**	32	93	53 : 47	76	48	8 : 92	99	>99
	103	93	51 : 49	43	10			
**2d**	30	96	79 : 21	78	75	5 : 95	97	>99
	108	96	79 : 21	54	49			
**2e**	30	96	88 : 12	92	90	5 : 95	99	>99
	105	96	88 : 12	83	77			
**2f**	32	99	83 : 18	97	89	8 : 92	99	>99
	92	99	83 : 17	94	77			

^*a*^Energy values obtained with the M06-2X/6-311G(2d,2p) level of theory were used for the obtainment of all the results reported in the table.

^*b*^Expressed as *syn* : *anti* ratio.

It is evident that the CHP provides only a poor qualitative agreement with the experimental data of Table 1. Indeed, the dr is predicted in favor of the *anti* diastereoisomer and the enantioselectivity is always predicted to be higher than 97%, in accordance with the recent report by Rzepa *et al.*7*a* (a discussion about the difficulty of the prediction of low levels of enantioselectivity can be found at page S14 of the ESI†). Moreover, no information about the reversibility of the process can be obtained.

When applying the MTSA, as expected (*vide infra*), the reaction times relative to the simulation are shorter than the corresponding experimental values. The evolution times of the kinetics equations were chosen in such a way that the selectivity of the first reaction (Table 3) matched with the corresponding experimental data (Table 1), that is *ca.* 30 h. Given the reaction time ratio of 6 d *versus* 21 d (experimental times of Table 1), the second measurement of the simulated reaction has been done at *ca.* 90/105 h (Table 3, entry 2).^20^ The same reaction times have been selected for the other reactions in order to obtain a comparison between reactions characterized by different substrates. The conversions are well reproduced, and respect the activity/stability trend reported above (compare Tables 1 and 3). Most importantly, a correct qualitative description of the dr is obtained, since the *syn* diastereoisomer is predicted to be favored, especially when compared with the CHP results in the left columns of Table 3. Analogously, the MTSA ee results are in good agreement with the experimental ones. These results are explained by the ability of the MTSA to take into account the equilibrating nature of the system, correctly predicting the erosion of the stereoselectivity. Moreover, the relative trends observed for the experimental results can be, in most cases, recognized also in the reported simulation outcomes: (i) all the products are affected by racemization, (ii) products characterized by electron-donating groups present higher ee erosion, and (iii) the *anti* diastereoisomers present faster racemization.

## Conclusions

In conclusion, new mechanistic insight into the proline catalyzed aldol reaction is presented. RPKA has been used to prove the reversibility of the reaction and to obtain a rate law that integrates the previously reported power law by Blackmond *et al.*5*a* by taking into account the reaction reversibility. This makes the obtained kinetics law applicable to a wider range of substrates, even when the reaction does not provide quantitative conversions. LFERs have been used to rationalize the dependence of the reaction rates and of the final reaction conversion on the aldehyde activation. The analysis of the stereochemistry of the reaction has supported the evidence of the equilibrating process, which affects both diastereo- and enantioselectivity. This strong evidence makes the CHP unsuitable as a model for the computational rationalization of the experimentally observed stereochemical outcome and enabled us to introduce a MTSA for the elaboration of the computed energies that is based on the inclusion of both kinetics and thermodynamics in the description of the process. The use of this MTSA allowed us to computationally predict key features of the proline catalyzed aldol reaction and to compute realistic stereoselectivities for the first time.

## Supplementary Material

Supplementary information

## References

[cit1] List B., Lerner R. A., Barbas III C. F. (2000). J. Am. Chem. Soc..

[cit2] Sigman M. S., Jacobsen E. N. (1998). J. Am. Chem. Soc..

[cit3] Ahrendt K. A., Borths C. J., MacMillan D. W. C. (2000). J. Am. Chem. Soc..

[cit4] Allemann C., Gordillo R., Clemente F. R., Cheong P. H.-Y., Houk K. N. (2004). Acc. Chem. Res..

[cit5] Zotova N., Broadbelt L. J., Armstrong A., Blackmond D. G. (2009). Bioorg. Med. Chem. Lett..

[cit6] Bahmanyar S., Houk K. N. (2001). J. Am. Chem. Soc..

[cit7] Armstrong A., Boto R. A., Dingwall P., Contreras-Garcia J., Harvey M. J., Mason N. J., Rzepa H. S. (2014). Chem. Sci..

[cit8] Schmid M. B., Zeitler K., Gschwind R. M. (2011). J. Org. Chem..

[cit9] Blackmond D. G. (2005). Angew. Chem., Int. Ed..

[cit10] (a) Anslyn E. V. and Dougherty D. A., Modern Physical Organic Chemistry, University Science Books, Mill Valley, CA, 2006.

[cit11] Where *k* is the rate constant describing the forward kinetic process defined in eqn (2) as *k*_F_ and *K* is the thermal equilibrium constant calculated from the final conversions

[cit12] Curtin D. Y. (1954). Rec. Chem. Prog..

[cit13] Seeman J. I. (1983). Chem. Rev..

[cit14] The free rotation of the C–N bond of enamine **4**, becomes important only at the TS level. In other words, since the two enamine conformers are converting at a higher speed with respect to the stereodetermining step of the reaction, only the most stable conformer (s-*trans* enamine) can be taken in consideration

[cit15] Steinfeld J. I., Francisco J. S. and Hase W. L., Chemical Kinetics and Dynamics, Prentice Hall, Upper Saddle River, NJ, 2nd edn, 1998.

[cit16] Wheeler S. E., Moran A., Pieniazek S. N., Houk K. N. (2009). J. Phys. Chem. A.

[cit17] Hubin P. O., Jacquemin D., Leherte L., Vercauteren D. P. (2014). Chem. Phys..

[cit18] Wigner E. (1938). Trans. Faraday Soc..

[cit19] MATLAB 8.0 and Statistics Toolbox 8.1, The MathWorks, Inc., Natick, Massachusetts, United States.

[cit20] Since the differential equations are solved by a numerical method, only discrete reaction times are available. Thus, the simulation times may differ from different reactions accordingly with the availability of similar time values in the numerical solution

