# Supplementary material for: Kinetics versus thermodynamics in the proline catalyzed aldol reaction
Source: Chem Sci. 2016 May 6;7(8):5421–7. doi: 10.1039/c6sc01328g (PMC6021756; doi:10.1039/c6sc01328g)
Supplement: Supplementary file 1 [file SC-007-C6SC01328G-s001.pdf]

Electronic Supplementary Material (ESI) for Chemical Science.
This journal is © The Royal Society of Chemistry 2016

Kinetics vs Thermodynamics in the Proline Catalyzed Aldol Reaction

Manuel Orlandi, Michele Ceotto* and Maurizio Benaglia*

Supporting Information

Index

General Information....S2
Importance of the equilibration in the reaction kinetics....S2
Experimental details for RPKA....S3
"Same excess" experiment....S3
Determination of the reaction order in [**2**]....S3
Determination of the reaction order in [**1**]....S3
Determination of the reaction order in [**3**]....S6
Rationalization of the observed first reaction order in [**3**]....S6
Fitting of the profiles relative to different substrates....S7
General procedure for the proline catalyzed aldol reaction....S9
Retro-aldol reaction....S10
CHP Calculations....S12
Relative stability of reagents and products....S13
Correlation between errors and energies....S14
Computed energies....S15
Geometries of reactions involving 2a-c....S17
References....S27

General Information

Reactions were monitored by analytical thin-layer chromatography (TLC) using silica gel 60 F254 pre-coated glass plates (0.25 mm thickness) and visualized using UV light. Flash chromatography was carried out on silica gel (230-400 mesh). ^{1}H-NMR spectra were recorded on spectrometers operating at 300 MHz (Bruker Fourier 300 or AMX 300). Proton chemical shifts are reported in ppm (δ) with the solvent reference relative to tetramethylsilane (TMS) employed as the internal standard ($CDCl_3$ δ(1H)= 7.26 ppm). ^{13}C-NMR spectra were recorded on 300 MHz spectrometers (Bruker Fourier 300 or AMX 300) operating at 75 MHz, with complete proton decoupling. Enantiomeric excesses determinations were performed with Chiral Stationary Phase HPLC analysis on an Agilent 1200 series HPLC instrument.

Importance of the equilibration in the reaction kinetics

Equation (2), which is demonstrated below, is very similar to the power kinetic law previously reported by Blackmond et al. [Ref. 5a in the text]. However, the two equations differ in the inclusion of the backward process because when taking $k_{\mathrm{B}}^{obs} = 0$ the reaction rate found by Blackmond is reproduced. Thus, the Blackmond's rate law can be safely applied only for proline catalyzed aldol reactions presenting near to quantitative conversion, and it provides only a partial description of the process for reactions that do not proceed to completeness. Hence, we need a reversible kinetic law for the description of other systems, where the backward process was found to be important.

A demonstration of these statements can be obtained as follow. By fitting the experimental *d*[**3f**]/*dt* vs [**2f**] graphical law with the Blackmond's equation $d[\mathbf{3f}]/dt = k[\mathbf{2f}]^{0.9}[\mathbf{1}]^{0.6}$, a k=0.14 is found with a R^2=0.97; indeed, the experimental curve appears as a line with intercept near to zero, while the power law presents a similar line with a zero intercept (Figure S1a). However, when the reaction reversibility becomes important as for **2a**, the power law still presents a zero intercept while the experimental data present a clearly different profile (Figure S1b).

NOTE: the power exponents 0.9 (for aldehyde **2f**) and 0.6 (for cyclohexanone **1**) were obtained by following the procedure previously described by Blackmond et al. [Ref. 5a in the text].The same dependence of the reaction rate on [**1**] was found by performing the reaction with different starting concentrations of **1** (see below).

Figure S1. Blue circles: fitted power law, red crosses: experimental data.

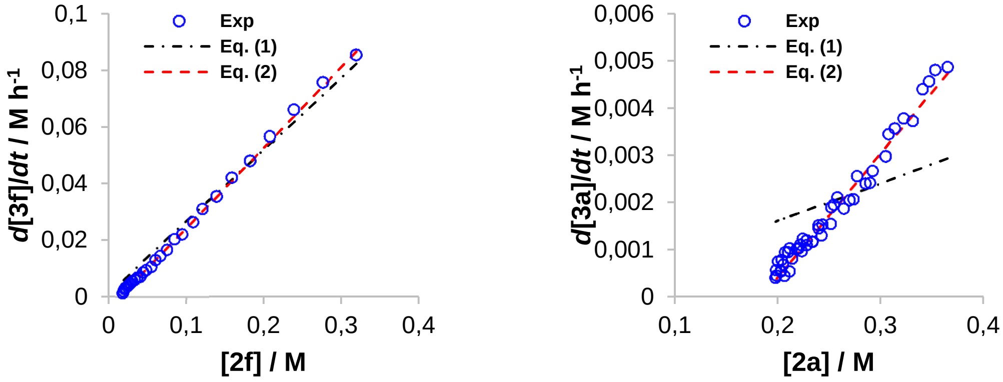

For the results obtained by fitting the experimental profiles of all the aldehydes **2a-2f** see below.

Experimental details for RPKA

A vial was charged with L-proline **5** (0.09 mmol), water, DMSO-d_6 (one 0.75 mL phial) and cyclohexanone **1** respectively. The vial was sealed and put in an ultrasound bath for 3 min. The aldehyde **2** was then added to the fine suspension and the resulting mixture was charged in a NMR tube that was sealed and immediately put in the NMR probe at 300 K. The ^{1}H NMR spectra were acquired with a delay between 20 min and 2 h depending on the substrate. The spectra (especially in the presence of water) did not show any substantial amount of side products.
In Table S1 the list of the performed experiments is reported.

Table S1.

run	substrate	$[1]_0$ / M	$[2]_0$ / M	[exc] / M	$[H_2O]_0$ / M	[5] / M
1	**2f**	0.4	0.4	0.0	0.73	0.12
2	**2f**	0.64	0.4	0.24	0.73	0.12
3	**2f**	1.33	0.4	0.93	0.73	0.12
4	**2f**	1.93	0.4	1.53	0.73	0.12
4-50%	**2f**	1.73	0.2	1.53	0.73	0.12
5	**2f**	3.0	0.4	2.6	0.73	0.12
6	**2a**	1.93	0.4	1.53	0.73	0.12
7	**2b**	1.93	0.4	1.53	0.73	0.12
8	**2c**	1.93	0.4	1.53	0.73	0.12
9	**2d**	1.93	0.4	1.53	0.73	0.12
10	**2e**	1.93	0.4	1.53	0.73	0.12

"Same excess" experiment

The curve's overlay in Figure S2 (**run 4** *vs* **run 4-50%**) confirms the absence of catalyst's deactivation in the used reaction conditions.

Figure S2.

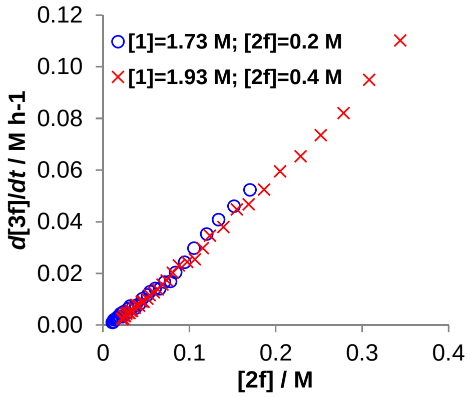

Determination of the reaction order in [2]

The graphical rate law *d*[**3f**]/*dt* vs [**2f**] depicted in Figure S1 and Figure S2 exhibits a linear dependence of *d*[**3f**]/*dt* on the aldehyde concentration [**2f**]. This means that the reaction is first order in aldehyde **2**.

Determination of the reaction order in [1]

The plots "*d*[**3f**]/*dt* vs [**2f**]" for **runs 1-5** present non superimposable lines (Figure S3), meaning a reaction order different from 1 for cyclohexanone **1**. Thus a mathematical analysis is required to get the cyclohexanone reaction order.
Curve's fitting have been performed both on the "[**2**] vs t" and "*d*[**3f**]/*dt* vs [**2f**]" profiles using the Origin® software and the functions reported in equations (S1) and (S2) in order to obtain the angular coefficient of the showed lines. The intercept *b* has a fundamental physical meaning and its presence in equation (S1) allows to include the experimentally observed reversibility into the kinetic law (NOTE: *b* may seem negligible in the case of aldehyde **2f**, but it is surely much more important for other aldehydes **2a-2e** for which the final equilibrium was established at relevant concentration of unreacted aldehyde, see Figure S4). Indeed, by considering that $[\mathbf{2f}]_0=[\mathbf{2f}]+[\mathbf{3f}]$, equation (S1) is related to equation (S3) with $g = b/[\mathbf{2}]_0$ and $f = a - g$.
These two parameters (*f* and *g*) bring the information of how strong the forward and backward processes are, and by

fitting reaction profiles obtained with "different excess" reactions, we obtained different f and g values. Thus, from the observed changes in f and g we can find the dependence of both the forward and backward processes on other kinetically relevant species.
In Table S2 the obtained values of f and g for the exponential fitting are reported for each run.

$$d[\mathbf{3}]/dt = a[\mathbf{2}] - b \qquad \text{(S1)}$$

$$[\mathbf{2}] = \frac{b+(a[\mathbf{2}]-b)e^{-at}}{a} \qquad \text{(S2)}$$

$$d[\mathbf{3}]/dt = f[\mathbf{2}] - g[\mathbf{3}] \qquad \text{(S3)}$$

Figure S3.

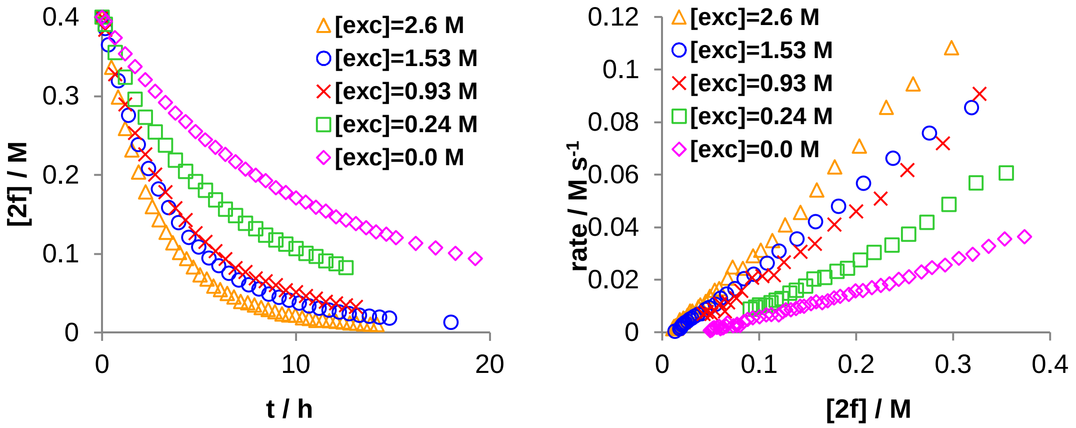

Table S2.

run	substrate	f	g	run	substrate	f	g
1	**2f**	0.0918	0.0109	6	**2a**	0.0149	0.0096
2	**2f**	0.1688	-[a]	7	**2b**	0.0580	0.0113
3	**2f**	0.2576	-[a]	8	**2c**	0.0754	0.0091
4	**2f**	0.3110	0.0095	9	**2d**	0.1384	0.0090
5	**2f**	0.3531	0.0091	10	**2e**	0.1474	0.0119

a) Since the reaction profile has not been acquired up to reaction equilibrium, the g value of these runs may be affected by a consistent error. Indeed, while f substantially depends on the starting reaction rate and can be efficiently determined even with incomplete reaction profiles, g is dependent on the final conversion and requires a complete reaction profile to be confidently determined.

From Table S2 it can be observed that while g presents only a slight decrement dependence on $[\mathbf{1}]_0$ (starting concentration of **1**) such that it can be considered roughly constant (g=9.8±1.1•10^{-3} h-1), f presents a clear dependence on $[\mathbf{1}]_0$ (Figure S3). Interestingly, the plot f vs $[\mathbf{1}]_0$ presents a profile similar to that one of a pre-equilibrating kinetics suggesting the presence of an activated intermediate that is due to the combination between the catalyst **5** and the substrate **1**, in analogy with enzymatic catalysis. Such a reactive species can be easily identified as the enamine intermediate **6**. Indeed, considering Scheme S1, we can express the concentration of enamine **6** as in equation (S7), where the steady state approximation and the assumption k_1, k_{-1} >> k_F, k_B, that takes into account the rate limiting step of the reaction, were used.

Scheme S1

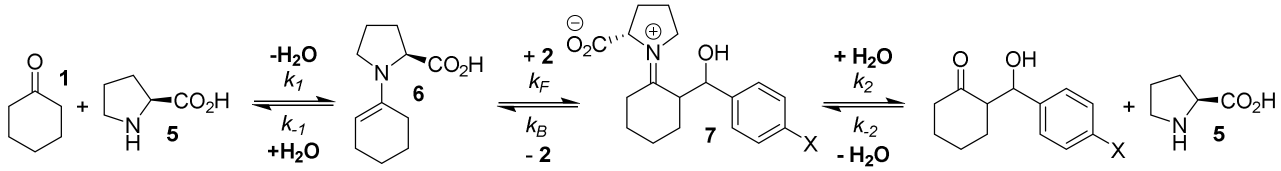

$$\frac{d[\mathbf{6}]}{dt} = [\mathbf{1}][\mathbf{5}]k_1 - [\mathbf{6}][\mathbf{H_2O}]k_{-1} = 0 \qquad \text{(S4)}$$

(***Steady state approximation* and k_1, k_{-1} >> k_F, k_B**)

Since $[\mathbf{5}]_{tot} = [\mathbf{5}] + [\mathbf{6}] + [\mathbf{7}]$

$$[\mathbf{1}][\mathbf{5}]_{tot}k_1 - [\mathbf{1}][\mathbf{7}]k_1 = [\mathbf{1}][\mathbf{6}]k_1 + [\mathbf{6}][\mathbf{H_2O}]k_{-1} \qquad \text{(S5)}$$

$$[\mathbf{6}] = \frac{[\mathbf{1}][\mathbf{5}]_{tot} - [\mathbf{1}][\mathbf{7}]}{[\mathbf{1}] + \frac{k_{-1}}{k_1}[\mathbf{H_2O}]} \qquad \text{(S6)}$$

Since it was shown that proline **5** present a quite low affinity for ketones,[1] **5** can be considered the resting state of the catalyst. Thus, [**5**]$_{tot}$ >> [**7**] and equation (S6) can be simplified in equation (S7).

$$[\mathbf{6}] = \frac{[\mathbf{1}][\mathbf{5}]_{tot}}{[\mathbf{1}] + \frac{k_{-1}}{k_1}[\mathbf{H_2O}]} \qquad \text{(S7)}$$

By fitting *f* vs [**1**]$_0$ with a function (S8) of the form of equation (S7) ([**H$_2$O**] is a constant amount in the reaction since it is not consumed), a good correlation is obtained (R^2>0.99) that validates equation (S7) (Figure S4).

$$f = \frac{m\,[\mathbf{1}]}{[\mathbf{1}]+n} \qquad (m = 0.565,\ n = 1.687) \qquad \text{(S8)}$$

Figure S4.

[**1**]$_0$ / M	[excess] / M	*f* / h^{-1}
3.0	2.6	0.361
1.93	1.53	0.311
1.33	0.89	0.246
0.64	0.24	0.168
0.4	0.0	0.096

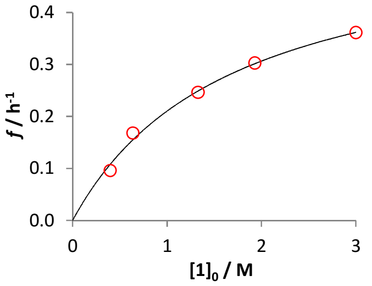

This dependence of the reaction rate on [**1**] was proved to be in excellent agreement with the reaction order of 0.58 previously reported by Blackmond.[5a] Indeed, by fitting equation (S8) (with *m* = 0.565, *n* = 1.687) with an exponential function of general formula *f* = s [**1**]^q, an exponent q=0.57 was found (Figure S5).[5a]

Figure S5.

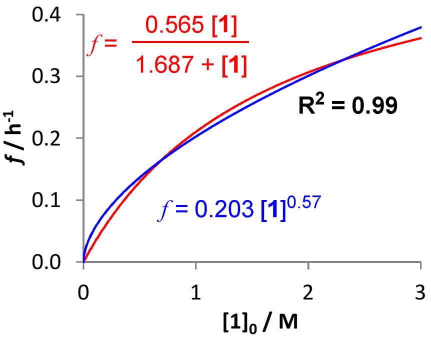

The dependence of the reaction on water has already been widely studied and found, by Blackmond et al.,[5b] to have a power exponent of -0.7. This dependence is compatible with the presence of the term [**H$_2$O**]k_{-1}/k_1 at the denominator of equation (S7). Thus, by substituting equation (S7) into (S3), expression (S9) is obtained.

$$d[\mathbf{3}]/dt = \frac{k_F[\mathbf{1}][\mathbf{5}]_{tot}}{[\mathbf{1}] + \frac{k_{-1}}{k_1}[\mathbf{H_2O}]}[\mathbf{2}] - g[\mathbf{3}] \qquad \text{(S9)}$$

The retro-aldol reaction of *anti*-**3a** to give back **1** and **2a** presents a first order kinetics in **5**. Indeed, by plotting the obtained initial rates of five different reactions against their different loading [**5**]$_{tot}$, a first order kinetics with respect to the catalyst was deduced (see the chapter “Retro-aldol reaction”). This means that proline **5** can catalyze both forward and backward aldol reactions. Thus, the parameter *g* of equation (S9) can be written as a new observed rate constant k_B^{obs} multiplied for the catalyst concentration [**5**]. Once again, since **5** is the catalyst’s resting state we can write [**5**]$_{tot}$ ≈ [**5**], and the final kinetic law will have the form reported in equation (S10).

$$d[\mathbf{3}]/dt = \frac{k_F[\mathbf{1}][\mathbf{2}][\mathbf{5}]_{tot}}{[\mathbf{1}]+\frac{k_{-1}}{k_1}[\mathbf{H_2O}]} - k_B^{obs}[\mathbf{3}][\mathbf{5}]_{tot} \quad \text{(S10)}$$

Determination of the reaction order in [3]

[**3**] was found to appear in the kinetic law thanks to the reaction's reversibility. However, the simple first order dependence deduced from equations (S1) and (S3) needs confirmation since, from Scheme S1, one may expect a more complex dependence of the reaction's rate on [**3**] due to the presence of the intermediate **7**. Thus the retro aldol reaction of **3a** was monitored through ^{1}H NMR providing the profile -*d*[**3a**]/*dt* vs [**3a**] depicted in Figure S6. Conditions: $[\mathbf{1}]_0$ = 0 M, $[\mathbf{2a}]_0$ = 0 M, $[\mathbf{3a}]_0$ = 0.075 M, [**5**] = 0.0375 M, [$\mathbf{H_2O}$] = 0.26 M.

Figure S6.

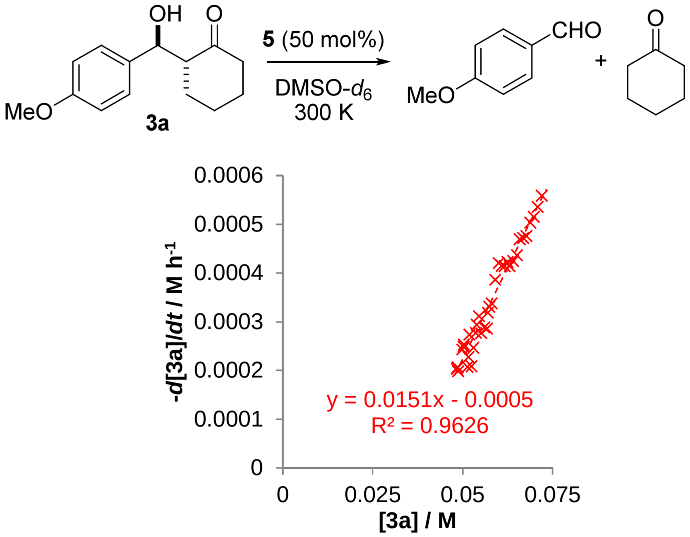

The linear dependence of the reaction rate on the ketol's concentration [**3a**] is a confirmation of the first order dependence of the reaction's rate to equilibrium on the product **3**. This experimental observation supports the validity of equation (S10).

Rationalization of the observed first reaction order in [3]

By considering Scheme S1 one may apply the stationary state approximation to intermediate **7** leading to equation (S11).

$$\frac{d[\mathbf{7}]}{dt} = [\mathbf{3}][\mathbf{5}]k_{-2} - [\mathbf{7}][\mathbf{H_2O}]k_2 = 0 \quad \text{(S11)}$$

(***Steady state approximation* and k_2, k_{-2} >> k_F, k_B**)

Since $[\mathbf{5}]_{tot} = [\mathbf{5}] + [\mathbf{6}] + [\mathbf{7}]$

$$[\mathbf{3}][\mathbf{5}]_{tot}k_{-2} - [\mathbf{3}][\mathbf{6}]k_{-2} = [\mathbf{3}][\mathbf{7}]k_{-2} + [\mathbf{7}][\mathbf{H_2O}]k_2 \quad \text{(S12)}$$

$$[\mathbf{7}] = \frac{[\mathbf{3}][\mathbf{5}]_{tot} - [\mathbf{3}][\mathbf{6}]}{[\mathbf{3}]+\frac{k_2}{k_{-2}}[\mathbf{H_2O}]} \quad \text{(S13)}$$

Since [**6**] is small due to the catalyst's resting state **5**, $[\mathbf{5}]_{tot}$ >> [**6**] and expression (S13) can be simplified in equation (S14).

$$[\mathbf{7}] = \frac{[\mathbf{3}][\mathbf{5}]_{tot}}{[\mathbf{3}]+\frac{k_2}{k_{-2}}[\mathbf{H_2O}]} \quad \text{(S15)}$$

The rationalization of the experimentally observed first order dependence of **3** can be explained considering the zwitterionic iminium **7** as a poorly stable species characterized by an high dissociation constant given by the ratio k_2/k_{-2}. Indeed, by hypothesizing $[\mathbf{H_2O}]k_2/k_{-2}$ >> [**3**] equation (S15) can be simplified into equation (S16).

$$[\mathbf{7}] = \frac{[\mathbf{3}][\mathbf{5}]_{tot}}{\frac{k_2}{k_{-2}}[\mathbf{H_2O}]} \quad \text{(S16)}$$

Since the addition of **6** to **2** is the rate determining step of the reaction, the reaction progress can be expressed accordingly with Scheme S1 as in equation (S17).

$$-\frac{d[\mathbf{2}]}{dt} = k_{\mathrm{F}}[\mathbf{2}][\mathbf{6}] - k_{\mathrm{B}}[\mathbf{7}] \qquad \text{(S17)}$$

Introducing equations (S7) and (S16) into (S17) one obtains:

$$-\frac{d[\mathbf{2}]}{dt} = \frac{k_{\mathrm{F}}[\mathbf{2}][\mathbf{1}][\mathbf{5}]_{tot}}{[\mathbf{1}]+\frac{k_{-1}}{k_1}[\mathbf{H_2O}]} - \frac{k_{\mathrm{B}}[\mathbf{3}][\mathbf{5}]_{tot}}{\frac{k_2}{k_{-2}}[\mathbf{H_2O}]} \qquad \text{(S18)}$$

Because [$\mathbf{H_2O}$] is constant during the reaction, one experimentally find an observed constant $k_{\mathrm{B}}^{\mathrm{obs}} = k_{\mathrm{B}}k_{-2}$ / [$\mathbf{H_2O}$]k_2 that once included in equation (S185) leads to the experimentally obtained kinetic law (S10).

By considering that [**2**]$_0$=[**2**]+[**3**], one can conveniently express equation (S10) as a function of the reagents concentration as reported in the text (see equation (1)).

$$\frac{d[3]}{dt} = \left[\frac{k_{\mathrm{F}}[\mathbf{1}]}{[\mathbf{1}]+\frac{k_{-1}}{k_1}[\mathbf{H_2O}]} + k_{\mathrm{B}}^{obs}\right][\mathbf{2}][\mathbf{5}]_{tot} - k_{\mathrm{B}}^{obs}[\mathbf{5}]_{tot}[\mathbf{2}]_0 \qquad (2)$$

If k_{B}^{obs} is set to zero, one recovers Blackmond rate expression [Ref. 5a in the text].

Fitting of the profiles relative to different substrates

In Figure S7 a series of graphs showing experimental and fitted profiles with the derived kinetic law (2) and with the previously reported power law (1) for all the substrates **2a-2f** are reported. All the exponential fits provided R^2>0.95. Experimental points are reported as blue circles, while the fitting obtained through equations (1) and (2) are reported as black dashed lines and red dashed lines respectively.

Figure S7.

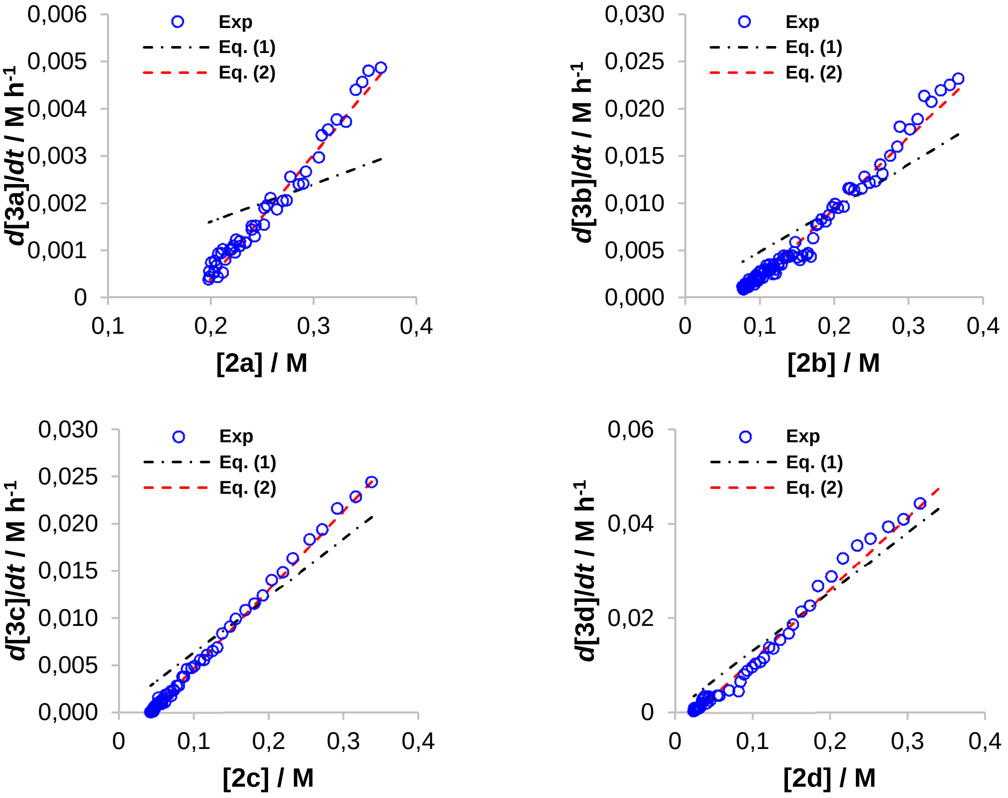

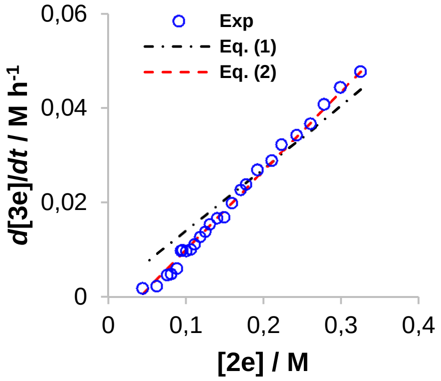

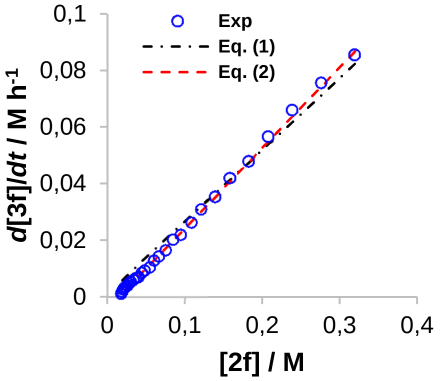

From the fitting reported in Figure S7, the constants k_F and k_B^{obs} were determined and reported in Figure 2 (see the main text of the paper).

General procedure for the proline catalyzed aldol reaction

To a stirred solution of L-proline (0.45 mmol) in DMSO (3.75 mL) the cyclohexanone (0.75 mmol) and water (2.75 mmol) were added. After 20 minutes the aldehyde (2 mmol) was added and the reaction was stirred at 25 °C. After 144 h an aliquot of the reaction mixture was taken, poured into 5 mL of a NH_4Cl saturated solution and extracted with AcOEt (3x10). The collected organic phases were washed with brine, dried with Na_2SO_4 and then concentrated in vacuum to afford the crude product that was analyzed by ^{1}H-NMR for the determination of the conversion and of the *syn:anti* ratio. It was then purified by flash column chromatography (Hex/AcOEt 7:3 for **2a** and **2e** or 8:2 for others ketols) to afford the pure ketol as mixture of diastereoisomer.

2-[hydroxy(4-methoxyphenyl)methyl]cyclohexanone (2a):

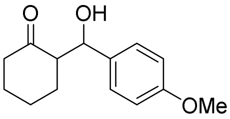

^{1}H-NMR data were in agreement with those previously reported in the literature. The enantiomeric excesses were evaluated by CSP-HPLC on a Chiralpak AD column (hexane:isopropanol 95:5; flow rate 0.5 mL/min; λ=230 nm): t_{syn} (minor)= 35.9 min, t_{syn} (major)= 41.9 min; t_{anti} (minor)= 63.3 min, t_{anti} (major)= 68.5 min.

2-[hydroxy(phenyl)methyl]cyclohexanone (2b):

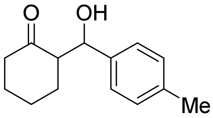

^{1}H-NMR data were in agreement with those previously reported in the literature. The enantiomeric excesses were evaluated by CSP-HPLC on a Chiracel AS column (hexane:isopropanol 9:1; flow rate 1.0 mL/min; λ=220 nm): t_{syn} (major)= 13.1 min, t_{syn} (minor)= 15.7 min; t_{anti} (major)= 16.5 min, t_{anti} (minor)= 20.3 min.

2-[hydroxy(phenyl)methyl]cyclohexanone (2c):

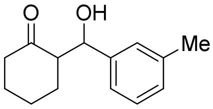

^{1}H-NMR data were in agreement with those previously reported in the literature. The enantiomeric excesses were evaluated by CSP-HPLC on a Chiracel AD column (hexane:isopropanol 9:1; flow rate 0.8 mL/min; λ=210 nm): t_{syn} (min)= 9.6 min, t_{syn} (major)= 11.3 min; t_{anti} (minor)= 14.9 min, t_{anti} (major)= 16.4 min.

2-[hydroxy(phenyl)methyl]cyclohexanone (2d):

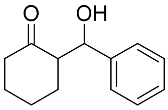

^{1}H-NMR data were in agreement with those previously reported in the literature. The enantiomeric excesses were evaluated by CSP-HPLC on a Chiracel OD-H column (hexane:isopropanol 98:2; flow rate 0.8 mL/min; λ=250 nm): t_{syn} (major)= 15.8 min, t_{syn} (minor)= 18.4 min; t_{anti} (major)= 22.5 min, t_{anti} (minor)= 39.9 min.

2-[hydroxy (4-chlorophenyl)methyl]cyclohexanone (2e):

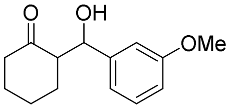

^{1}H-NMR data were in agreement with those previously reported in the literature. The enantiomeric excesses were evaluated by CSP-HPLC on a Chiralpak AD column (hexane:isopropanol 9:1; flow rate 0.5 mL/min; λ=230 nm): t_{syn} (min)= 23.7 min, t_{syn} (major)= 26.4 min; t_{anti} (minor)= 38.8 min, t_{anti} (major)= 43.0 min.

2-[hydroxy (4-chlorophenyl)methyl]cyclohexanone (2f):

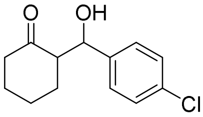

^{1}H-NMR data were in agreement with those previously reported in the literature. The enantiomeric excesses were evaluated by CSP-HPLC on a Chiralpak AD column (hexane:isopropanol 9:1; flow rate 0.5 mL/min; λ=230 nm): t_{syn} (min)= 16.6 min, t_{syn} (major)= 19.5 min; t_{anti} (minor)= 25.3 min, t_{anti} (major)= 29.6 min.

The reaction outcomes for different reaction times are summarized in Table S3.

Table S3.

entry	substrate	t (d)	conv. (%)	*syn:anti*	*ee*$_{syn}$ (%)	*ee*$_{anti}$ (%)
1	**2a**	6	58	55:45	59	76
2	**2a**	21	68	55:45	34	48
3	**2b**	6	87	53:47	73	79
4	**2b**	21	87	56:44	54	54
5	**2c**	6	90	51:49	64	60
6	**2c**	21	90	56:44	41	19
7	**2d**	6	95	53:47	77	77
8	**2d**	21	95	58:42	69	60
9	**2e**	6	95	53:47	82	83
10	**2e**	21	95	58:42	72	63
11	**2f**	6	98	48:52	87	84
12	**2f**	21	98	58:42	84	78

Retro-aldol reaction

The desired racemic ketols **2** (0.2 mmol) synthesized according to a previously reported procedure,[2] 1,3-dinitrobenzene (0.1 mmol, *internal standard*) and *rac*-proline (0.1 mmol) was dissolved in 2 mL of DMSO-d_6. An NMR tube was filled with the resulting mixture and periodic ^{1}H-NMR analyses were performed. The results are shown in Table S4.

Table S4. Time evolution of ketol:aldehyde ratios of the retro-aldol reaction.

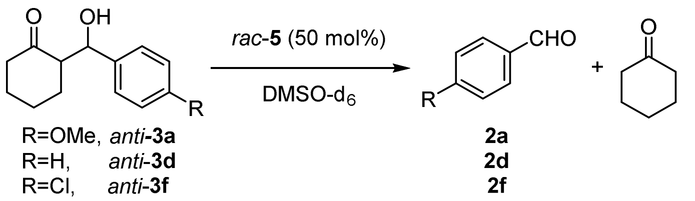

t (h)	3a:2a	3d:2d	3f:2f
**0**	>99:1	>99:1	>99:1
**4**	96:3	98:2	98:2
**20**	86:14	95:5	97:3
**28**	83:17	94:6	96:4
**48**	76:24	93:7	96:4
**68**	69:31	93:7	96:4
**92**	62:38	92:8	96:4
**503**	28:72	90:10	95:5

In order to prove the involvement of proline in the retro-aldol reaction, we performed the reaction with *rac*-*anti*-**3a** and with different loadings of (*S*)-proline **5** in DMSO-d_6 (0.075M) at 35°C, we found a linear dependency of the reaction rate with respect to the catalyst's concentration. A similar study was previously reported by List and Houk.[3]

Table S5.

0 mol%		10 mol%		20 mol%		30 mol%		40 mol%		50 mol%	
t [h]	2a [%]	t [h]	2a [%]	t [h]	2a [%]	t [h]	2a [%]	t [h]	2a [%]	t [h]	2a [%]
0,2	98,1	0,3	98,7	0,4	98,7	0,5	98,7	0,6	98,5	0,7	98,7
8,1	98,0	8,2	95,1	8,2	92,6	8,3	91,2	8,4	89,3	8,5	88,2
23,6	98,0	23,6	89,0	23,7	84,3	23,8	81,7	23,9	78,0	23,9	75,6
32,2	98,0	32,3	87,2	32,4	82,6	32,4	79,0	32,5	74,5	32,5	70,9
47,4	98,2	47,5	84,8	47,6	80,1	47,6	75,5	47,7	70,5	47,8	67,5
56,0	98,0	55,7	84,4	55,7	79,0	55,8	74,7	55,8	69,6	55,9	66,3
72,3	98,0	72,4	84,0	72,5	78,5	72,5	72,7	72,6	67,7	72,7	64,0

By interpolating the obtained curves with third order polynomials, we obtained the expressions:

[10 mol% (*S*)-pro] $^{10}[\mathbf{2a}] = -2E\text{-}05t^3 + 0.0065t^2 – 0.553t + 98.972$ $R^2=0.99$
[20 mol% (*S*)-pro] $^{20}[\mathbf{2a}] = -7E\text{-}05t^3 + 0.0137t^2 – 0.8833t + 98.951$ $R^2=0.99$
[30 mol% (*S*)-pro] $^{30}[\mathbf{2a}] = -1E\text{-}04t^3 + 0.0166t^2 – 1.0701t + 99.14$ $R^2=0.99$
[40 mol% (*S*)-pro] $^{40}[\mathbf{2a}] = -1E\text{-}04t^3 + 0.0203t^2 – 1.3061t + 99.147$ $R^2=0.99$
[50 mol% (*S*)-pro] $^{50}[\mathbf{2a}] = -1E\text{-}04t^3 + 0.0245t^2 – 1.5191t + 99.583$ $R^2=0.99$

These equations give an excellent description of the concentration of **3a** during time (t). Hence, their derivatives give us the value of the reaction rate in each point of the selected time range (0-72h). The derivate expressions are:

[10 mol% (*S*)-pro] $d^{10}[\mathbf{2a}]/dt = -6E\text{-}05t^2 + 0.013t – 0.553$
[20 mol% (*S*)-pro] $d^{20}[\mathbf{2a}]/dt = -2.1E\text{-}04t^2 + 0.027t – 0.8833$
[30 mol% (*S*)-pro] $d^{30}[\mathbf{2a}]/dt = -3E\text{-}04t^2 + 0.0332t – 1.0701$
[40 mol% (*S*)-pro] $d^{40}[\mathbf{2a}]/dt = -0.0003t^2 + 0.0406t – 1.3061$
[50 mol% (*S*)-pro] $d^{50}[\mathbf{2a}]/dt = -0.0003t^2 + 0.049t – 1.5191$

The initial rates associated to the five profiles can be obtained simply by calculating the limit of these polynomials for $t \rightarrow 0$. We obtain:

[10 mol% (*S*)-pro] $(d^{10}[\mathbf{2a}]/dt)_0 = -0.553$
[20 mol% (*S*)-pro] $(d^{20}[\mathbf{2a}]/dt)_0 = -0.8833$
[30 mol% (*S*)-pro] $(d^{30}[\mathbf{2a}]/dt)_0 = -1.0701$
[40 mol% (*S*)-pro] $(d^{40}[\mathbf{2a}]/dt)_0 = -1.3061$
[50 mol% (*S*)-pro] $(d^{50}[\mathbf{2a}]/dt)_0 = -1.5191$

The unite of measure of such initial rates is: [% h^{-1}], and give us the percentage variation of **3a** with respect to **2a** at the very beginning of the reaction.
Proline is a catalyst and the only reagent is **3a**, thus we can hypothesize the first order kinetics:

$d[\mathbf{3a}]/dt = -[\mathbf{3a}]k'$ where $k' = k_B[\mathbf{pro}]$.

And for t=0: $(d[\mathbf{2a}]/dt)_0 = -[\mathbf{2a}]_0[\mathbf{pro}]k$

Since $[\mathbf{2a}]_0$ and k are common to each reaction profile, we will obtain the relative initial reaction rates to be directly proportional to the relative amount of catalyst. Hence, by plotting the proline loading against the calculated initial rates we should obtain a linear relationship (Figure S8).

Figure S8 linear dependence of the retro-aldol reaction on the proline loading.

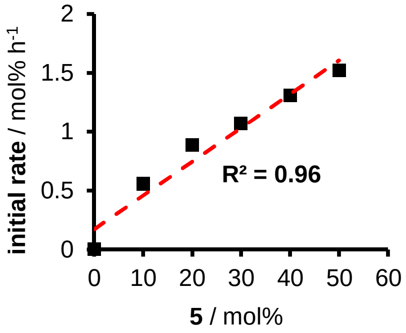

A further proof of the involvement of proline in the retro-aldol reaction has been found when *rac*-*anti*-**2a** was mixed with (*S*)-proline (30 mol%) and cyclohexanone (4 eq.) at 40°C. The appearance of *syn*-**2a** was detected. After 72 h the crude mixture was extracted with AcOEt/NH_4Cl ss. and directly injected in a Chiral Stationary Phase HPLC revealing a 1:3 *syn:anti* ratio and 53% *ee* toward the *RS* enantiomer for *anti*-**2a**. *SR* is the favored product of the (*S*)-proline catalyzed reaction leading to compounds **2**, hence, a kinetic resolution of the racemic mixture occurred, where the (*S*)-proline preferentially reacted with ***SR*-2a**.

CHP Calculations

In Table S6, stereoselections obtained with different computational set-ups within the CHP approach are reported. Independently from the chosen computational method, the calculated *ee* was >99% for all the computations, and the wrong *anti*-isomer was always predicted as the major one, thus missing an appropriate qualitative prediction of *dr*. For sake of comparison with literature data[4] Table S6 reports the widely employed B3LYP functional failure results as well.

Table S6.

**Method**	**2a**		**2d**		**2f**	
	**syn:anti**	**ee[a]**	**syn:anti**	**ee[a]**	**syn:anti**	**ee[a]**
B3LYP/6-31G(d,p)	2:98	>99	<1:99	>99	1:99	>99
B3LYP/6-311+G(3df,3pd)[b]	<1:99	>99	<1:99	>99	<1:99	>99
M06-2X/6-31G(d,p)	23:77	>99	8:92	>99	12:88	>99
M06-2X/6-311+G(d,p)	-	-	3:97	>99	-	-
M06-2X/6-311G(2d,2p)	3:97	>99	8:92	>99	15:85	>99
M06-2X/TZVP	-	-	1:99	>99	-	-
M06-2X/cc-PVTZ	-	-	2:98	>99	-	-
M06-2X/6-311+G(3df,3pd)[c]	11:89	>99	4:96	>99	6:94	>99
M06-2X/6-311+G(3df,3pd)[c,d]	4:96	>99	2:98	>99	3:97	>99
M06-HF/6-31G(d,p)	-	-	9:91	>99	-	-
M06-HF/6-311+G(d,p)	-	-	20:80	>99	-	-
M06-HF/TZVP	-	-	1:99	>99	-	-
M05-2X/6-31G(d,p)	-	-	3:97	>99	-	-
M05-2X/6-311+G(d,p)	-	-	1:99	>99	-	-
M05-2X/TZVP	-	-	<1:99	>99	-	-
MPW1PW91/6-31G(d,p)	2:98	>99	1:99	>99	1:99	>99
MPW1PW91/6-311+G(3df,3pd)[e]	<1:99	>99	1:99	>99	<1:99	>99

[a] ee [%] of the anti isomer. [b] Single Point Energy calculation at B3LYP/6-31G(d,p) geometries and Gibbs free energy corrections. [c] Single Point Energy calculation at M06-2X/6-31G(d,p) geometries and Gibbs free energy corrections. [d] IEFPCM-DMSO solvent reaction field. [e] Single Point Energy calculation at MPW1PW91/6-31G(d,p) geometries and Gibbs free energy corrections.

Relative stability of reagents and products

Our approach also requires a good description of the thermodynamic properties such as the relative stability of reagents and products; this fact allows to obtain good predictions regarding the yields and the stereoselectivity. M06-2X and MPW1PW91 functionals were reported to better fit the thermodynamics description of aldol, Mannich and α-aminoxylation proline-catalyzed reactions.[5] Also, Hubin and coworkers recently reported M06-2X as an efficient functional for the computational treatment of proline-catalyzed propionaldehyde's self-condensation.[6] A further check is provided by our calculations of Gibbs free energy of reaction $\Delta_R G$ for the aldol addition of cyclohexanone to aldehyde **2d** with different computational set up (Table S7). The reported energies are relative to the single point calculation on the gas phase M06-2X/6-311G(2d,2p) geometry and thermal correction to Gibbs free energy unless specified.

Table S7.

functional	basis set	solvation model	$\Delta_R G_{syn}$ kcal/mol	$\Delta_R G_{anti}$ kcal/mol	Pop. (%)[a]	*syn:anti*[b]
**M06-2X**	**6-311G(2d,2p)**	**-**	**-1.42**	**-0.62**	**73**	**79:21**
M06-2X	6-311+G(d,p)	-	-0.94	-0.62	89	63:37
M06-2X	6-311G(2d,2p)	CPCM	1.02	1.45	21	67:33
M06-2X	6-311G(2d,2p)	IEFPCM	1.03	1.45	21	67:33
M06-2X	6-311G(2d,2p)	IPCM	0.86	1.04	29	58:42
M06-2X[c]	6-311G(2d,2p) [c]	PCM[c]	1.02	1.32	24	62:38
M06-2X	6-311+G(3df,2p)	IPCM	2.72	2.51	2	41:59
M06-2X	6-311+G(2df,2pd)	IPCM	2.49	2.32	3	43:57
M06-2X	cc-PVTZ	IPCM	2.45	2.41	3	49:51
M06-2X-GD3	6-311G(2d,2p)	IPCM	0.23	0.41	54	57:43
M06-2X-GD3	cc-PVTZ	IPCM	1.82	1.79	9	49:51
M05-2X	6-311+G(d,p)	-	-2.01	-1.97	98	52:48
M05-2X	6-311G(2d,2p)	IPCM	0.29	0.49	51	58:42
M05-2X	6-311+G(3df,2p)	IPCM	2.14	1.93	6	41:59
M05-2X	6-311+G(2df,2pd)	IPCM	1.86	1.70	9	43:57
M05-2X	cc-PVTZ	IPCM	1.66	1.65	11	50:50
M06-HF	6-311+G(d,p)	-	-8.92	-7.84	>99	86:14
M06-HF	6-311G(2d,2p)	IPCM	-5.46	-5.30	>99	57:43
M06-HF	6-311+G(3df,2p)	IPCM	-3.67	-4.09	>99	33:67
M06-HF	6-311+G(2df,2pd)	IPCM	-3.78	-3.95	>99	43:57
M06-HF	6-311++G(3df,3pd)	IPCM	-4.33	-4.71	>99	35:65
M06-HF[d]	6-311++G(3df,3pd)	IPCM	-4.53	-4.93	>99	34:66
M06-HF	cc-PVTZ	IPCM	-3.41	-3.44	>99	49:51
M06	6-311G(2d,2p)	IPCM	6.69	5.95	<1	22:78
M06L	6-311G(2d,2p)	IPCM	11.35	10.7	<1	26:74
B3LYP	6-311G(2d,2p)	PCM	14.3	13.90	<1	35:65
BMK	6-311G(2d,2p)	PCM	7.78	7.50	<1	38:62
wB97XD	6-311G(2d,2p)	PCM	3.14	2.80	1	36:64
wB97XD	6-311+G(2df,2pd)	PCM	4.83	3.93	<1	18:82
wB97XD	6-311++G(3df,3pd)	PCM	4.90	3.96	<1	17:83
wB97XD	cc-PVTZ	PCM	5.13	4.49	<1	25:75
LC-wPBE	6-311G(2d,2p)	PCM	2.91	2.71	2	42:58
LC-wPBE	6-311+G(2df,2pd)	PCM	4.76	3.94	<1	20:80
LC-wPBE	6-311++G(3df,3pd)	PCM	4.81	3.94	<1	19:81
LC-wPBE	cc-PVTZ	PCM	4.82	4.27	<1	28:72

a) Calculated according to the formula: $100(\exp(-\Delta_R G_{anti}/RT)+\exp(-\Delta_R G_{syn}/RT))/(1+\exp(-\Delta_R G_{anti}/RT)+\exp(-\Delta_R G_{syn}/RT))$. b)The *syn:anti* ratio was calculated according to the formula $\exp(-(\Delta_R G_{syn}-\Delta_R G_{anti})/RT)$. c) Geometry and freq analysis with M06-2X/6-311G(2d,2p)[PCM=DMSO]. d) Geometry and freq analysis with M06-HF/6-311G(2d,2p).

Correlation between errors and energies.

It is important to point out that the correct evaluation of low level of stereoslectivity (as in our case, where the enantiomeric excesses are between 50% and 90%) is extremely difficult. According to the formula on the left (Figure S3), for small ΔΔG (e.g. from 0 to 1.5 kcal/mol) the *ee* exhibits a more sloped curve, which flattens increasing these values (from 1.5 kcal/mol or 85% ee). For a graphical proof see Figure S3: a small error of ±0.2 kcal/mol in the evaluation of a ΔΔG = 0.5 kcal/mol lead to an error of ±15% *ee*. On the other hand, the error decreases to ±1.1% *ee* when ΔΔG is 2.3 kcal/mol. In the same way, it can be pointed out that the evaluation of very low or very high chemical yields is simpler with respect to the evaluation of yields values around 50%, for which the relative $\Delta_R G$ is 0.0 kcal/mol (Figure S3, right).

Figure S9.

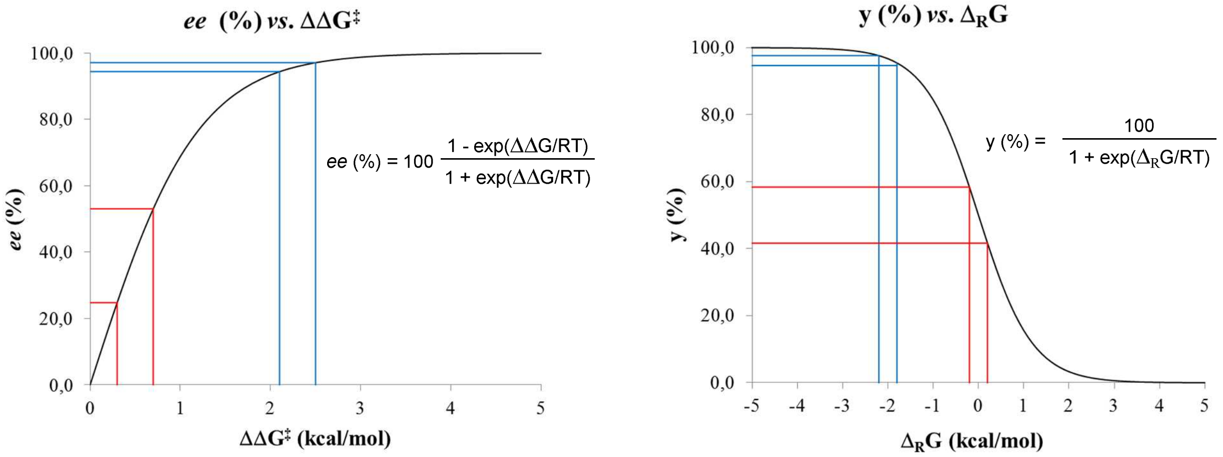

Computed energies

Aldehyde 2a:

	G_{rel} (kcal/mol)	*k* forward	*k* backward	
Pro Cy=O 4-OMe	0,00			
TS1 4-OMe	15,14	4,92416E+01	2,07839E+05	
En h2o 4-OMe	4,94			
h2o				$\Delta\Delta G^{\ddagger}$ (kcal/mol)
ss	21,15	8,14003E+00	2,86905E-03	2,41
rr	24,21	4,60197E-02	1,62202E-05	5,47
sr	20,11	4,66301E+01	2,89820E-02	1,37
rs	24,99	1,24642E-02	7,74688E-06	6,25
Pro				
syn	0,24			
anti	0,57			

Aldehyde 2b:

	G_{rel} (kcal/mol)	*k* forward	*k* backward	
Pro Cy=O 4-Me	0,00			
TS1 4-Me	15,14	4,92416E+01	2,07839E+05	
En h2o 4-Me	4,94			
h2o				$\Delta\Delta G$ (kcal/mol)
ss	20,40	2,86363E+01	1,64477E-03	1,66
rr	23,45	1,67464E-01	9,61853E-06	4,71
sr	18,77	4,51114E+02	3,39973E-02	0,03
rs	24,08	5,76376E-02	4,34374E-06	5,34
Pro				
syn	-1,95			
anti	-0,51			

Aldehyde 2c:

	G_{rel} (kcal/mol)	*k* forward	*k* backward	
Pro Cy=O 3-Me	0,00			
TS1 3-Me	15,14	4,92416E+01	2,07839E+05	
En h2o 3-Me	4,94			
h2o				$\Delta\Delta G$ (kcal/mol)
ss	20,07	4,97902E+01	2,85977E-03	1,43
rr	23,47	1,60183E-01	9,20034E-06	4,83
sr	18,64	5,61598E+02	4,23237E-02	0,00
rs	22,98	3,69699E-01	2,78616E-05	4,34
Pro				
syn	-0,84			
anti	-0,68			

Aldehyde 2d:

	G_{rel} (kcal/mol)	*k* forward	*k* backward	
Pro Cy=O PhCHO	0,00			
TS1 PhCHO	15,14	4,92416E+01	2,07839E+05	
En h2o PhCHO	4,94			
h2o				ΔΔG (kcal/mol)
ss	20,50	2,43896E+01	5,21278E-04	1,76
rr	22,93	4,01953E-01	8,59093E-06	4,19
sr	18,74	4,73772E+02	3,90853E-02	0,00
rs	23,64	1,20522E-01	9,94284E-06	4,90
Pro				
syn	-1,42			
anti	-0,62			

Aldehyde 2e:

	G_{rel} (kcal/mol)	*k* forward	*k* backward	
Pro Cy=O 3-OMe	0,00			
TS1 3-OMe	15,14	4,92416E+01	2,07839E+05	
En h2o 3-OMe	4,94			
h2o				ΔΔG (kcal/mol)
ss	20,64	1,90378E+01	2,90477E-04	1,73
rr	23,63	1,21958E-01	1,86083E-06	4,72
sr	18,91	3,54420E+02	3,99930E-02	0,00
rs	24,62	2,31611E-02	2,61352E-06	5,71
Pro				
syn	-1,62			
anti	-0,44			

Aldehyde 2f:

	G_{rel} (kcal/mol)	*k* forward	*k* backward	
Pro Cy=O 3-OMe	0,00			
TS1 3-OMe	15,14	4,92416E+01	2,07839E+05	
En h2o 3-OMe	4,94			
h2o				ΔΔG (kcal/mol)
ss	20,23	3,83740E+01	1,44463E-04	1,49
rr	23,40	1,81820E-01	6,84478E-07	4,66
sr	18,74	4,70904E+02	9,37990E-03	0,00
rs	23,52	1,48161E-01	2,95121E-06	4,78
Pro				
syn	-2,45			
anti	-1,47			

Geometries of reactions involving 2a-c

Geometries and energies obtained at the M06-2X/6-311G(2d,2p) level of theory of the involved species are reported below for substrate **2d**.

Cyclohexanone:

C	-0.3885010	-1.2792760	0.3704150
C	-0.3885010	1.2792760	0.3704150
C	0.9972610	1.2579610	-0.2905980
C	1.7727200	0.0000000	0.0953280
C	0.9972610	-1.2579610	-0.2905980
H	-0.9936370	2.1210400	0.0409620
H	-0.2617560	-1.3487280	1.4557700
H	-0.9936370	-2.1210400	0.0409620
H	0.8745810	1.2850300	-1.3765210
H	1.5517340	2.1540880	-0.0119220
H	2.7508450	0.0000000	-0.3867580
H	1.9496070	0.0000000	1.1753140
H	0.8745810	-1.2850300	-1.3765210
H	1.5517340	-2.1540880	-0.0119220
H	-0.2617560	1.3487280	1.4557700
C	-1.1469000	0.0000000	0.0769780
O	-2.2627920	0.0000000	-0.3745960

Zero-point correction=	0.152152 (Hartree/Particle)
Thermal correction to Energy=	0.158571
Thermal correction to Enthalpy=	0.159516
Thermal correction to Gibbs Free Energy=	0.121837
Sum of electronic and zero-point Energies=	-309.690371
Sum of electronic and thermal Energies=	-309.683951
Sum of electronic and thermal Enthalpies=	-309.683007
Sum of electronic and thermal Free Energies=	-309.720686

Proline:

N	0.5191730	1.2701750	-0.0207730
C	1.5796290	0.6776720	0.8044310
C	-0.1030030	0.1264680	-0.7211490
H	1.1245470	0.2737220	1.7106680
H	2.3015020	1.4387440	1.0935080
C	2.1612310	-0.4568740	-0.0402990
C	0.9056690	-1.0480120	-0.6999580
H	-0.3998570	0.4262800	-1.7233800
C	-1.3599220	-0.1877380	0.0696270
H	2.7136440	-1.1915790	0.5422680
H	2.8347850	-0.0440480	-0.7935090
H	0.5036610	-1.8558710	-0.0920190
H	1.0959690	-1.4323640	-1.6992230
O	-1.4143400	-0.9164930	1.0210940
O	-2.4230890	0.4976600	-0.3804980
H	-3.1531670	0.3008540	0.2198260
H	0.9425200	1.8746110	-0.7134170

Zero-point correction=	0.146473 (Hartree/Particle)
Thermal correction to Energy=	0.153866
Thermal correction to Enthalpy=	0.154811
Thermal correction to Gibbs Free Energy=	0.113870
Sum of electronic and zero-point Energies=	-400.969230
Sum of electronic and thermal Energies=	-400.961837
Sum of electronic and thermal Enthalpies=	-400.960893
Sum of electronic and thermal Free Energies=	-401.001834

TS1: TS for the addition of the *L*-proline to the cyclohexanone.

N	0.1724410	0.6654670	-0.1649620
C	1.2843590	-0.0313030	-0.8736360
C	2.1130940	-1.0735050	-0.0787230
O	3.0908960	-1.4917120	-0.6468560
H	-0.3886700	1.1008130	-0.8895460
C	0.8083490	1.7398110	0.6485210
C	2.2039800	1.1350020	-1.2433860
H	3.1800270	0.7785550	-1.5556030
C	2.2215080	1.9415560	0.0616730
H	1.7572180	1.7141030	-2.0540150
H	2.4542520	2.9917530	-0.0938710
H	2.9671450	1.5275560	0.7390330
H	0.8214630	1.3891430	1.6792820
H	0.1930990	2.6348080	0.5894380
H	0.8838810	-0.5542400	-1.7401770
H	0.8362330	-1.0886580	1.5043690
C	-1.0111500	-0.3082330	0.8846120
O	-0.3599540	-0.5745860	1.9449430
O	1.7608180	-1.4418110	1.1167200
C	-1.3525970	-1.4591290	-0.0578390
H	-2.0041210	-2.1059220	0.5370460
H	-0.4558490	-2.0437800	-0.2641320
C	-2.0885310	-1.0414400	-1.3315600
H	-1.4193840	-0.4914140	-2.0023430
H	-2.3953900	-1.9309920	-1.8818500
C	-2.1473880	0.6955080	1.0584340
H	-2.8330960	0.1960700	1.7487450
H	-1.7644040	1.5660320	1.5910900
C	-2.9033480	1.0717420	-0.2151570
H	-2.2980840	1.7279800	-0.8511850
H	-3.7867760	1.6551600	0.0456380
C	-3.3044000	-0.1722080	-1.0079250
H	-4.0091310	-0.7591190	-0.4117770
H	-3.8228490	0.1119570	-1.9239470

Zero-point correction=	0.300471 (Hartree/Particle)
Thermal correction to Energy=	0.313496
Thermal correction to Enthalpy=	0.314440
Thermal correction to Gibbs Free Energy=	0.261008
Sum of electronic and zero-point Energies=	-710.658922
Sum of electronic and thermal Energies=	-710.645897
Sum of electronic and thermal Enthalpies=	-710.644953
Sum of electronic and thermal Free Energies=	-710.698385

Water:

O	0.0000000	0.0000000	0.1176480
H	0.0000000	0.7564990	-0.4705920
H	0.0000000	-0.7564990	-0.4705920

Zero-point correction=	0.021693 (Hartree/Particle)
Thermal correction to Energy=	0.024528
Thermal correction to Enthalpy=	0.025472
Thermal correction to Gibbs Free Energy=	0.004059
Sum of electronic and zero-point Energies=	-76.394755
Sum of electronic and thermal Energies=	-76.391920
Sum of electronic and thermal Enthalpies=	-76.390976
Sum of electronic and thermal Free Energies=	-76.412390

Enamine:

H	-1.2528330	1.7691610	2.2147660
N	-0.6070970	-0.7290280	-0.4495460
C	-1.5038780	0.4025400	-0.6728190
C	-1.3272110	1.4996060	0.3653890
O	-1.1321600	2.6577740	0.1256230
C	-1.3120790	-1.7624670	0.2880960
C	-2.9265920	-0.2085620	-0.5808190
H	-3.4656940	0.2312450	0.2561770
C	-2.6987050	-1.7098800	-0.3413960
H	-3.4969620	-0.0101160	-1.4841520
H	-2.6827090	-2.2447160	-1.2896940
H	-3.4669440	-2.1500600	0.2899610
H	-1.3518400	-1.5482520	1.3635410
H	-0.8130720	-2.7201640	0.1435990
H	-1.3133450	0.8721680	-1.6361620
C	0.7524930	-0.4845220	-0.2105800
C	1.4645480	-1.1517870	0.7037690
C	1.3743370	0.5418160	-1.1322470
C	2.9410450	-0.9501100	0.9181670
H	0.9751450	-1.8871800	1.3280970
C	2.8973400	0.4441240	-1.1533660
H	0.9712930	0.3870980	-2.1353580
H	1.0712980	1.5484050	-0.8297180
C	3.4436700	0.3344330	0.2659890
H	3.4970640	-1.8049180	0.5167200
H	3.1513020	-0.9337420	1.9894940
H	3.1941540	-0.4433820	-1.7186890
H	3.3158810	1.3095310	-1.6673880
H	3.0995380	1.1943620	0.8472860
H	4.5337030	0.3584100	0.2650280
O	-1.4111030	1.0227510	1.6234030

Zero-point correction=	0.274075 (Hartree/Particle)
Thermal correction to Energy=	0.286999
Thermal correction to Enthalpy=	0.287943
Thermal correction to Gibbs Free Energy=	0.233764
Sum of electronic and zero-point Energies=	-634.261937
Sum of electronic and thermal Energies=	-634.249013
Sum of electronic and thermal Enthalpies=	-634.248069
Sum of electronic and thermal Free Energies=	-634.302248

Benzaldehyde (2d):

C	-1.7266960	1.0545020	-0.0000430
C	-0.3582770	1.2849200	0.0000040
C	0.5269930	0.2137140	0.0000210
C	0.0471670	-1.0941420	-0.0000070
C	-1.3168900	-1.3237430	-0.0000520
C	-2.2026190	-0.2493140	-0.0000700
H	-2.4183270	1.8850220	-0.0000580
H	0.0282650	2.2970230	0.0000290
H	0.7617290	-1.9059850	0.0000050
H	-1.6968380	-2.3357790	-0.0000720
H	-3.2685150	-0.4319720	-0.0001060
C	1.9873670	0.4679810	0.0000650
O	2.8219100	-0.3960520	0.0000740
H	2.2761300	1.5366020	0.0001010

Zero-point correction=	0.110832 (Hartree/Particle)
Thermal correction to Energy=	0.117085
Thermal correction to Enthalpy=	0.118030

Thermal correction to Gibbs Free Energy=	0.080340
Sum of electronic and zero-point Energies=	-345.413990
Sum of electronic and thermal Energies=	-345.407736
Sum of electronic and thermal Enthalpies=	-345.406792
Sum of electronic and thermal Free Energies=	-345.444482

TS-H-SR-F: favored TS for the addition of the enamine to benzaldehyde leading to the *SR*-product.

H	-1.0326010	-0.6841570	1.5369930
N	-1.6877770	-0.3125890	-0.8228740
C	-2.9825810	-0.4640460	-0.1370710
C	-2.9880310	-0.0441270	1.3436250
O	-4.0026050	0.4373790	1.7830300
C	-1.3228570	-1.5339850	-1.5737570
C	-3.2683960	-1.9633810	-0.2740810
H	-2.7611920	-2.4939360	0.5335000
C	-2.6297340	-2.3183540	-1.6137200
H	-4.3330180	-2.1702250	-0.2155010
H	-3.2567190	-1.9703040	-2.4356100
H	-2.4589480	-3.3845420	-1.7391570
H	-0.5479550	-2.0706180	-1.0318550
H	-0.9534530	-1.2517910	-2.5584410
H	-3.7507860	0.1244370	-0.6390170
C	0.9050770	-0.0997990	0.7397540
O	0.1198960	-1.0912390	1.0000670
C	-0.9172560	0.7508640	-0.7286760
C	0.4681680	0.6534210	-0.9954990
C	-1.4693130	2.0042600	-0.1043200
C	1.2475750	1.9218160	-1.2870270
H	0.7698360	-0.1938120	-1.5992380
C	-0.7639750	3.2568100	-0.6258890
H	-2.5448280	2.0614050	-0.2670440
H	-1.3290770	1.9270890	0.9796310
C	0.7479310	3.1215600	-0.4860340
H	1.1621220	2.1507920	-2.3536340
H	2.3063800	1.7453530	-1.0999670
H	-1.0180320	3.4097560	-1.6779670
H	-1.1315620	4.1237440	-0.0780650
H	1.0018700	3.0034950	0.5706640
H	1.2448780	4.0282620	-0.8308830
H	0.7756830	0.8121080	1.3404080
C	2.3518790	-0.4256550	0.5174070
C	3.3460880	0.4889180	0.8460930
C	2.7106630	-1.6713900	0.0107470
C	4.6849000	0.1730070	0.6519000
H	3.0708110	1.4476870	1.2695370
C	4.0447430	-1.9865620	-0.1871520
H	1.9290950	-2.3900330	-0.1973750
C	5.0354130	-1.0629390	0.1291880
H	5.4520470	0.8879480	0.9157220
H	4.3180630	-2.9569870	-0.5785370
H	6.0767780	-1.3114840	-0.0218740
O	-1.9143820	-0.2784590	2.0400170

Zero-point correction=	0.385866 (Hartree/Particle)
Thermal correction to Energy=	0.404347
Thermal correction to Enthalpy=	0.405291
Thermal correction to Gibbs Free Energy=	0.339424
Sum of electronic and zero-point Energies=	-979.678291
Sum of electronic and thermal Energies=	-979.659810
Sum of electronic and thermal Enthalpies=	-979.658866
Sum of electronic and thermal Free Energies=	-979.724733

TS-H-SR-D: disfavored TS for the addition of the enamine to benzaldehyde leading to the *SR*-product.

H	-1.0886500	-0.7803950	1.3973900
N	-1.6847230	-0.1940930	-0.8847560
C	-3.0350680	-0.2811310	-0.3051780
C	-3.0727840	-0.2033700	1.2386540
O	-4.1326470	0.0867370	1.7346930
C	-1.3134450	-1.4565780	-1.5607050
C	-3.5593850	-1.6378660	-0.7946210
H	-4.2752580	-2.0536900	-0.0910600
C	-2.2854850	-2.4630990	-0.9635520
H	-4.0560860	-1.5123790	-1.7573220
H	-2.4186540	-3.3338430	-1.6006350
H	-1.9060030	-2.7861520	0.0061270
H	-0.2828130	-1.7132570	-1.3599180
H	-1.4697730	-1.3314790	-2.6359370
H	-3.6656390	0.5308750	-0.6569970
C	0.9047970	-0.1480660	0.7162760
O	0.1024400	-1.1404260	0.8838390
C	-0.8897820	0.8481970	-0.7353610
C	0.4976440	0.7526200	-0.9838460
C	-1.4348390	2.0720680	-0.0443280
C	1.3048770	2.0255640	-1.1528230
H	0.8032550	-0.0386160	-1.6552470
C	-0.6761390	3.3473500	-0.4112250
H	-2.4948950	2.1878080	-0.2594060
H	-1.3630510	1.8884950	1.0341500
C	0.8259860	3.1560280	-0.2477770
H	1.2337720	2.3552520	-2.1939950
H	2.3578780	1.8077930	-0.9771920
H	-0.8941480	3.6123880	-1.4490670
H	-1.0389130	4.1648800	0.2108040
H	1.0494560	2.9268380	0.7974300
H	1.3566530	4.0763890	-0.4911160
H	0.7720930	0.7243610	1.3717250
C	2.3497120	-0.4730570	0.4882620
C	3.3491180	0.4140280	0.8727920
C	2.7024270	-1.6943510	-0.0790070
C	4.6865520	0.0974470	0.6714640
H	3.0789810	1.3506530	1.3459440
C	4.0354100	-2.0096550	-0.2844040
H	1.9172260	-2.3965480	-0.3257900
C	5.0310410	-1.1119140	0.0858700
H	5.4573180	0.7905440	0.9793620
H	4.3033460	-2.9607310	-0.7239840
O	-1.9920820	-0.4707020	1.9109920
H	6.0714720	-1.3606830	-0.0711050

Zero-point correction=	0.386158 (Hartree/Particle)
Thermal correction to Energy=	0.404626
Thermal correction to Enthalpy=	0.405571
Thermal correction to Gibbs Free Energy=	0.339488
Sum of electronic and zero-point Energies=	-979.677759
Sum of electronic and thermal Energies=	-979.659290
Sum of electronic and thermal Enthalpies=	-979.658346
Sum of electronic and thermal Free Energies=	-979.724429

TS-H-RS: unique TS for the addition of the enamine to the benzaldehyde leading to the *RS*-product.

H	-0.4953020	-1.2890460	-1.1715220
N	-2.0358490	0.3118590	0.0561100
C	-2.1523560	-0.8945590	0.8928930
C	-1.3412730	-2.1465240	0.4910090

O	-1.2874210	-3.0258360	1.3163630
C	-3.1561940	0.4146320	-0.8988510
C	-3.6417550	-1.2384330	0.7454400
H	-3.8311120	-2.2739560	1.0112580
C	-3.9018820	-0.9058690	-0.7237730
H	-4.2358720	-0.5915100	1.3927830
H	-4.9571660	-0.8099940	-0.9656420
H	-3.4608550	-1.6708620	-1.3613040
H	-2.7578140	0.5663720	-1.9001030
H	-3.7973920	1.2558280	-0.6306910
H	-1.8779410	-0.6531620	1.9163240
C	0.9109220	0.4087540	-1.0025620
O	0.0267190	-0.2208880	-1.6968360
H	1.1211730	1.4294040	-1.3427650
C	2.1711090	-0.2690070	-0.5508770
C	3.3340340	0.4907280	-0.4406930
C	2.2230560	-1.6351350	-0.2768680
C	4.5291140	-0.0930250	-0.0446770
H	3.3055430	1.5460390	-0.6863570
C	3.4172100	-2.2157350	0.1219530
H	1.3356540	-2.2431400	-0.3830900
C	4.5703330	-1.4494330	0.2431620
H	5.4258100	0.5070700	0.0281290
H	3.4477690	-3.2753120	0.3352390
C	-1.1036310	1.2346340	0.1910780
C	0.1562210	0.9368280	0.7456330
C	-1.2890600	2.5579980	-0.4955280
C	0.9873210	2.0579650	1.3394430
H	0.2290870	-0.0058670	1.2784380
C	-0.5962150	3.6912090	0.2681060
H	-0.8719190	2.4553060	-1.5043230
H	-2.3466400	2.7800450	-0.6160930
C	0.8649070	3.3667460	0.5598170
H	2.0286760	1.7405080	1.3944650
H	0.6670460	2.2277130	2.3717130
H	-0.6803010	4.6140110	-0.3056070
H	-1.1241110	3.8536970	1.2113740
H	1.3216380	4.1771150	1.1281360
H	1.4133380	3.3003900	-0.3821340
H	5.4986050	-1.9103270	0.5518360
O	-0.8640660	-2.2262730	-0.7134100

Zero-point correction=	0.385335 (Hartree/Particle)
Thermal correction to Energy=	0.403870
Thermal correction to Enthalpy=	0.404815
Thermal correction to Gibbs Free Energy=	0.338301
Sum of electronic and zero-point Energies=	-979.669884
Sum of electronic and thermal Energies=	-979.651349
Sum of electronic and thermal Enthalpies=	-979.650405
Sum of electronic and thermal Free Energies=	-979.716918

TS-H-SS-D: disfavored TS for the addition of the enamine to benzaldehyde leading to the *SS*-product.

H	0.8033570	1.2451810	-0.9702680
N	1.9284980	-0.6977500	0.0243220
C	2.7856480	0.3975050	0.5086230
C	2.0761280	1.7573620	0.6252390
O	2.4473710	2.5068620	1.4947730
C	2.5612250	-1.4255360	-1.0975450
C	3.8977430	0.4617280	-0.5449380
H	3.5681260	1.0969660	-1.3685710
C	4.0174590	-0.9840990	-1.0183800
H	4.8120640	0.8747920	-0.1291700
H	4.5553190	-1.5810750	-0.2807820

H	4.5226030	-1.0813730	-1.9759490
H	2.0923080	-1.1136700	-2.0283930
H	2.4259380	-2.4957950	-0.9540780
H	3.1918200	0.1569740	1.4907660
C	-0.6889400	-0.3593350	-1.5470570
O	0.3292180	0.3941450	-1.8331660
C	0.7306290	-0.9632810	0.4927780
C	-0.1853440	-1.7200920	-0.2871330
C	0.2649120	-0.3041530	1.7589830
C	-1.3446540	-2.4030090	0.4215960
H	0.2933060	-2.3688930	-1.0101260
C	-0.6866470	-1.2191860	2.5340710
H	1.1161950	-0.0111500	2.3715280
H	-0.2563700	0.6190790	1.4845040
C	-1.8520980	-1.6517630	1.6527240
H	-1.0123460	-3.3985170	0.7319280
H	-2.1607700	-2.5648150	-0.2826420
H	-0.1385400	-2.0979640	2.8847610
H	-1.0436970	-0.6903480	3.4169140
H	-2.4223700	-0.7712340	1.3530080
H	-2.5292750	-2.2962840	2.2137160
H	-0.9338600	-1.0985210	-2.3197580
C	-1.9536240	0.2560100	-1.0181050
C	-3.1690450	-0.3834270	-1.2567950
C	-1.9538680	1.4650920	-0.3238020
C	-4.3545640	0.1296400	-0.7520060
H	-3.1863610	-1.2862240	-1.8548940
C	-3.1408040	1.9804250	0.1790370
H	-1.0301180	2.0094440	-0.1872730
C	-4.3402470	1.3079130	-0.0172050
H	-5.2888000	-0.3810200	-0.9414000
H	-3.1280740	2.9162750	0.7207990
H	-5.2617870	1.7120440	0.3788920
O	1.1644600	2.0289460	-0.2583910

Zero-point correction=	0.386141 (Hartree/Particle)
Thermal correction to Energy=	0.404245
Thermal correction to Enthalpy=	0.405190
Thermal correction to Gibbs Free Energy=	0.341236
Sum of electronic and zero-point Energies=	-979.676647
Sum of electronic and thermal Energies=	-979.658542
Sum of electronic and thermal Enthalpies=	-979.657598
Sum of electronic and thermal Free Energies=	-979.721552

TS-H-SS-F: favored TS for the addition of the enamine to benzaldehyde leading to the *SS*-product.

H	0.9049790	1.2511480	-0.8061450
N	1.8799520	-0.8097940	-0.0141110
C	2.7896870	0.1168440	0.6783350
C	2.2556190	1.5643620	0.7685350
O	2.7651090	2.2741070	1.5998120
C	2.4735110	-1.2837780	-1.2851370
C	4.0762580	0.0654520	-0.1551450
H	4.6212940	1.0023300	-0.0820470
C	3.5661790	-0.2613390	-1.5563540
H	4.7189210	-0.7364860	0.2098130
H	4.3368530	-0.6499580	-2.2170870
H	3.1146710	0.6186490	-2.0149200
H	1.7321020	-1.3017080	-2.0711210
H	2.8839650	-2.2837370	-1.1201240
H	2.9702430	-0.2014980	1.7010160
C	-0.6982260	-0.2022700	-1.5749470
O	0.3552230	0.5252510	-1.7694210

C	0.6704550	-1.1033020	0.4104570
C	-0.2690420	-1.7234220	-0.4566380
C	0.2159030	-0.5839220	1.7464260
C	-1.4775100	-2.4133980	0.1569550
H	0.1878100	-2.3266970	-1.2309290
C	-0.8103090	-1.5121940	2.3979170
H	1.0641090	-0.4245800	2.4078590
H	-0.2342160	0.4002090	1.5742610
C	-1.9757800	-1.7751670	1.4532010
H	-1.2075040	-3.4539360	0.3622350
H	-2.2843400	-2.4515880	-0.5752690
H	-0.3262070	-2.4554710	2.6650280
H	-1.1583320	-1.0577280	3.3249170
H	-2.4887910	-0.8352850	1.2445690
H	-2.7017050	-2.4385610	1.9239990
H	-0.9755820	-0.8444150	-2.4208640
C	-1.9283820	0.4156250	-0.9753630
C	-3.1797550	-0.1162720	-1.2794830
C	-1.8544670	1.5353010	-0.1476510
C	-4.3318040	0.4072830	-0.7111780
H	-3.2523650	-0.9418410	-1.9766720
C	-3.0068850	2.0599390	0.4204910
H	-0.8989660	2.0056880	0.0381740
C	-4.2451540	1.4883530	0.1557610
H	-5.2956070	-0.0199520	-0.9520170
H	-2.9386710	2.9257900	1.0649130
H	-5.1403360	1.8997050	0.6014110
O	1.3279450	1.9406310	-0.0590570

Zero-point correction=	0.386335 (Hartree/Particle)
Thermal correction to Energy=	0.404410
Thermal correction to Enthalpy=	0.405354
Thermal correction to Gibbs Free Energy=	0.341183
Sum of electronic and zero-point Energies=	-979.676779
Sum of electronic and thermal Energies=	-979.658704
Sum of electronic and thermal Enthalpies=	-979.657760
Sum of electronic and thermal Free Energies=	-979.721932

TS-H-RR-F: favored TS for the addition of the enamine to benzaldehyde leading to the *RR*-product.

H	-1.2095730	-1.5431890	-0.8834720
N	-1.8529760	0.7727060	-0.1220720
C	-2.8251540	-0.0139160	0.6622960
C	-2.7732250	-1.5586470	0.5402540
O	-3.4174050	-2.1587000	1.3703660
C	-2.4633680	1.2885490	-1.3656050
C	-4.1625500	0.4595820	0.0790880
H	-4.9518440	-0.2551730	0.2909260
C	-3.8283970	0.6081560	-1.4045730
H	-4.4343030	1.4263730	0.5062690
H	-4.5564430	1.1948440	-1.9588440
H	-3.7397570	-0.3755730	-1.8636890
H	-1.8252290	1.0433850	-2.2114360
H	-2.5757360	2.3722190	-1.2977960
H	-2.7204920	0.2301900	1.7157990
C	0.6064390	-1.1602270	-0.1051320
O	-0.1180650	-1.0857780	-1.1784030
H	0.3444120	-1.9529330	0.6025000
C	2.0724030	-0.9830930	-0.2749950
C	2.9556830	-1.3847920	0.7236260
C	2.5780720	-0.4491780	-1.4589330
C	4.3201650	-1.1871620	0.5753320
H	2.5695240	-1.8608640	1.6162390

C	3.9421580	-0.2507050	-1.6077220
H	1.8861960	-0.2198790	-2.2573390
C	4.8142320	-0.6026120	-0.5842480
H	4.9989310	-1.4982470	1.3573380
H	4.3296060	0.1672780	-2.5268990
C	-0.6250830	1.0442840	0.2614730
C	0.0512910	0.2556960	1.2207550
C	0.1657230	2.0609850	-0.5031390
C	1.1788530	0.8986650	2.0132320
H	-0.5917280	-0.3705310	1.8298740
C	1.1074480	2.8429340	0.4175940
H	0.7395830	1.5081870	-1.2562290
H	-0.4916400	2.7419180	-1.0375310
C	2.0066400	1.9063680	1.2161920
H	1.8302260	0.1283080	2.4243990
H	0.7342610	1.4058650	2.8756360
H	1.7049030	3.5286460	-0.1828450
H	0.5091630	3.4536040	1.0991270
H	2.6298810	2.4821250	1.9009850
H	2.6803840	1.3861750	0.5350610
H	5.8778700	-0.4469620	-0.7009360
O	-2.1514480	-2.0810250	-0.4614130

Zero-point correction=	0.384429 (Hartree/Particle)
Thermal correction to Energy=	0.403039
Thermal correction to Enthalpy=	0.403983
Thermal correction to Gibbs Free Energy=	0.337335
Sum of electronic and zero-point Energies=	-979.670962
Sum of electronic and thermal Energies=	-979.652351
Sum of electronic and thermal Enthalpies=	-979.651407
Sum of electronic and thermal Free Energies=	-979.718055

TS-H-RR-D: disfavored TS for the addition of the enamine to benzaldehyde leading to the *RR*-product.

H	-1.1879860	-1.6542490	-0.9604900
N	-1.8089910	0.7306510	-0.2092550
C	-2.8493110	-0.1103740	0.4418600
C	-2.6708350	-1.6423830	0.5173690
O	-3.2014840	-2.1812100	1.4635410
C	-2.3601800	1.3747150	-1.4111150
C	-4.1177240	0.1652550	-0.3876020
H	-4.2334600	-0.6273840	-1.1276920
C	-3.8428770	1.4896370	-1.0945720
H	-4.9999850	0.1714050	0.2453470
H	-4.0121960	2.3320190	-0.4224870
H	-4.4467960	1.6299330	-1.9877070
H	-2.1844080	0.7027450	-2.2538120
H	-1.8784280	2.3220050	-1.6189740
H	-2.9568290	0.2438880	1.4653970
C	0.6133660	-1.2212350	-0.2162970
O	-0.1452520	-1.1335610	-1.2675690
H	0.4405790	-2.0833080	0.4358650
C	2.0574710	-0.9206610	-0.4127610
C	3.0104860	-1.4018190	0.4813800
C	2.4745410	-0.1927770	-1.5256720
C	4.3521690	-1.0936230	0.3112650
H	2.6990990	-2.0282380	1.3074750
C	3.8148900	0.1178440	-1.6953930
H	1.7341880	0.0978590	-2.2581250
C	4.7545870	-0.3164820	-0.7676000
H	5.0848990	-1.4679900	1.0128820
H	4.1308890	0.6878780	-2.5585400
C	-0.6214070	0.9584120	0.3136650

C	-0.0352930	0.0203770	1.1971070
C	0.2154020	2.1108450	-0.1645870
C	1.0430540	0.4817740	2.1614730
H	-0.7370620	-0.6686850	1.6540530
C	1.0711010	2.6853630	0.9693290
H	0.8585760	1.7511060	-0.9739650
H	-0.4112280	2.8969580	-0.5756120
C	1.9244410	1.6070570	1.6216990
H	1.6581270	-0.3681470	2.4581690
H	0.5511590	0.8256780	3.0766210
H	1.6960510	3.4837220	0.5697220
H	0.4110510	3.1376670	1.7145700
H	2.5064850	2.0338700	2.4389490
H	2.6366350	1.2216080	0.8910050
O	-2.0907230	-2.2291790	-0.4714970
H	5.7999670	-0.0748360	-0.9009460

Zero-point correction=	0.384407 (Hartree/Particle)
Thermal correction to Energy=	0.403100
Thermal correction to Enthalpy=	0.404044
Thermal correction to Gibbs Free Energy=	0.337508
Sum of electronic and zero-point Energies=	-979.666276
Sum of electronic and thermal Energies=	-979.647583
Sum of electronic and thermal Enthalpies=	-979.646639
Sum of electronic and thermal Free Energies=	-979.713175

Ketol (*syn*-3d):

C	0.9487410	-1.0106910	-0.7213010
C	0.8881620	0.0606600	0.3826320
C	2.2526240	0.7174320	0.5006210
C	3.4090160	-0.2254660	0.7328880
C	3.4341580	-1.2845010	-0.3812910
C	2.0889940	-1.9993420	-0.4858790
C	-0.2082500	1.1133920	0.1498340
O	2.4130790	1.9085760	0.3600840
O	0.0059470	1.7929650	-1.0677910
C	-4.0722350	-0.7722730	0.1357620
C	-3.5480280	-0.2579020	-1.0409420
C	-2.3062620	0.3652620	-1.0444990
C	-1.5777480	0.4784640	0.1332360
C	-2.1123760	-0.0324150	1.3131670
C	-3.3505170	-0.6554420	1.3170980
H	1.0814430	-0.5041940	-1.6802060
H	-0.0090410	-1.5297790	-0.7640670
H	0.6970840	-0.4473920	1.3354180
H	4.3278340	0.3552350	0.7722330
H	3.2634390	-0.7231150	1.6959730
H	3.6566320	-0.7920060	-1.3313370
H	4.2390000	-1.9949040	-0.1937110
H	1.9020330	-2.5555630	0.4376700
H	2.1175100	-2.7299230	-1.2948550
H	-5.0395080	-1.2553010	0.1357170
H	-4.1082390	-0.3378590	-1.9627230
H	-1.8947350	0.7766850	-1.9546150
H	-1.5595310	0.0686490	2.2401200
H	-3.7558480	-1.0437070	2.2416340
H	0.8186840	2.2943570	-0.9469140
H	-0.1666330	1.8234350	0.9833500

Zero-point correction=	0.268216 (Hartree/Particle)
Thermal correction to Energy=	0.281101
Thermal correction to Enthalpy=	0.282045

Thermal correction to Gibbs Free Energy=	0.228306
Sum of electronic and zero-point Energies=	-655.127528
Sum of electronic and thermal Energies=	-655.114643
Sum of electronic and thermal Enthalpies=	-655.113699
Sum of electronic and thermal Free Energies=	-655.167438

Ketol (*anti*-3d):

C	0.9321490	-1.3042000	-0.2267550
C	0.9069540	0.1723980	0.2066300
C	2.2776980	0.7869870	-0.0404890
C	3.4549960	0.0304820	0.5305790
C	3.4286770	-1.4375050	0.0862690
C	2.0827610	-2.0750390	0.4162010
C	-0.1966970	1.0026570	-0.4700860
O	2.4268980	1.8059510	-0.6751790
O	-0.2005960	2.3185740	0.0423740
H	1.0290270	-1.3507100	-1.3170140
H	-0.0220520	-1.7650380	0.0241720
H	0.7335310	0.2109270	1.2894550
H	4.3666980	0.5418890	0.2299250
H	3.3762260	0.0731400	1.6215800
H	3.5993570	-1.4876530	-0.9921790
H	4.2447970	-1.9795340	0.5636120
H	1.9439530	-2.0897400	1.5011660
H	2.0650750	-3.1122020	0.0802680
H	0.6395430	2.7069520	-0.2210960
C	-1.5610670	0.4016340	-0.2364190
C	-2.1182940	-0.4788620	-1.1570450
C	-2.2602700	0.6936390	0.9307710
C	-3.3475070	-1.0751540	-0.9111780
H	-1.5862220	-0.6973830	-2.0751280
C	-3.4914330	0.1028610	1.1759910
H	-1.8385960	1.4029300	1.6297610
C	-4.0361320	-0.7865210	0.2585960
H	-3.7700900	-1.7579530	-1.6356770
H	-4.0302180	0.3407300	2.0831330
H	-4.9963660	-1.2454400	0.4501880
H	0.0039080	1.0126240	-1.5481230

Zero-point correction=	0.268081 (Hartree/Particle)
Thermal correction to Energy=	0.281036
Thermal correction to Enthalpy=	0.281980
Thermal correction to Gibbs Free Energy=	0.228030
Sum of electronic and zero-point Energies=	-655.126112
Sum of electronic and thermal Energies=	-655.113157
Sum of electronic and thermal Enthalpies=	-655.112213
Sum of electronic and thermal Free Energies=	-655.166163

References

1. B. List, L. Hoang, H. J. Martin *PNAS* **2004**, *101*, 5839.
2. M. Majewski, D. M. Gleave, *Tetrahedron Letters*, **1989**, *30*, 5681-5684.
3. L. Hoang, S. Bahmanyar, K. N. Houk, B. List, *J. Am. Chem. Soc*., **2003**, *125*, 16-17.
4. A. Armstrong, R. A. Boto, P. Dingwall, J. Contreras-Garcia, M. J. Harvey, N. J. Mason, H. S. Rzepa, *Chem. Sci*., **2014**, *5*, 2057-2071.
5. S. E. Wheeler, A. Moran, S. N. Pieniazek, K. N. Houk, *J. Phys. Chem. A* **2009**, *113*, 10376-10384.
6. P. O. Hubin, D. Jacquemin, L. Leherte, D. P. Vercauteren, *Chemical Physics* **2014**, *434*, 30-36.
